# Biodegradable cross-linked flavone networks featuring either ester or carbamate linkages for controlled delivery of anti-cancer agents

**DOI:** 10.1039/d6ra02770a

**Published:** 2026-05-12

**Authors:** Mohammed S. H. Alwaezi, Francesca Greco, Helen M. I. Osborn, Wayne Hayes

**Affiliations:** a Department of Chemistry, University of Reading Whiteknights Reading RG6 6AD UK w.c.hayes@reading.ac.uk m.s.h.alwaezi@pgr.reading.ac.uk; b Department of Chemistry, Faculty of Science, University of Zakho Zakho 42002 Iraq; c School of Pharmacy, University of Reading Whiteknights Reading RG6 6AD UK h.m.i.osborn@reading.ac.uk f.greco@reading.ac.uk

## Abstract

Flavonoids show broad preclinical anticancer activity, but their translation to the clinic is limited by poor aqueous solubility, rapid metabolism and limited systemic exposure after conventional administration. Here, the flavonoids quercetin, luteolin and apigenin were exploited as multifunctional building blocks to generate insoluble cross-linked flavonoid networks containing either ester (1–3) or carbamate (4–6) linkages, intended as degradable local depot matrices. Network formation was confirmed by FTIR and thermal analysis, and flavonoid loading was quantified by exhaustive degradation (pH 2.0, 60 °C), giving loadings of 28–36 wt/wt% (esters) and 32–44 wt/wt% (carbamates). Release of parent flavonoids was quantified by HPLC under aqueous conditions (PBS, 37 °C) at pH 7.4, 6.5 and 5.0. Ester networks showed comparatively higher baseline release at pH 7.4, whereas carbamate networks exhibited stronger pH-responsiveness (*e.g.*, cross-linked 4 : 6 wt/wt% at pH 7.4 *vs.* 24 wt/wt% at pH 5.0; 288 h). Extract-based MTT assays using MCF-7 cells showed that medium pre-exposed to ester networks for 24 h retained antiproliferative activity (*e.g.*, cross-linked 1 : 19% viability), whereas 5 min extracts were not cytotoxic, consistent with time-dependent release rather than rapid leaching. Cytocompatibility was also confirmed in the non-malignant fibroblast cell line 161BR, where all matched extract conditions were non-significantly different from the untreated control. Overall, incorporating flavonoids into degradable network architectures enables tunable release governed by linker chemistry, pH and flavonoid scaffold.

## Abstract 2

Cross-linked flavone networks have been generated that degrade under tumour-relevant conditions to demonstrate sustained release of the active flavonoid payloads.

## Graphical abstract

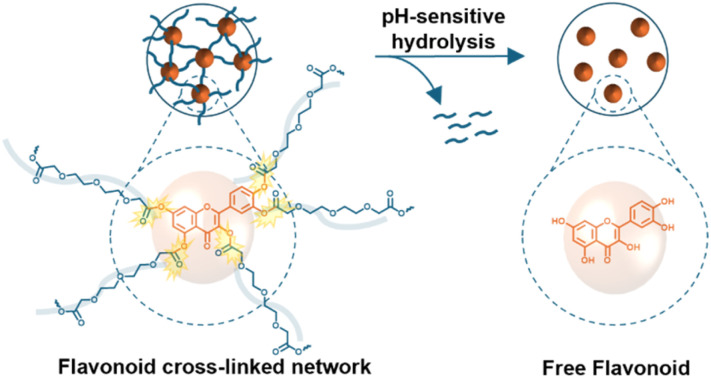

## Introduction

Localised, long-acting delivery strategies are increasingly being explored to overcome the limitations of systemic anticancer therapy,^1,2^ where many small molecules fail to sustain therapeutically relevant exposure at tumour sites because of rapid clearance,^3^ metabolic inactivation,^4^ chemical instability,^3^ and dose limiting off-target toxicity.^1,5^ These constraints motivate localised, long-acting delivery strategies that reduce reliance on systemic exposure by maintaining extracellular drug levels at or near the disease site.^6,7^ In particular, local depot formats that place a formulation adjacent to tumour tissue,^8^ including metastatic sites, or within/around a post-surgical resection cavity,^9^ can sustain exposure at sites of residual or recurrent disease while minimising systemic burden.^1,10^ Consequently, polymer-based implants and depot systems have attracted broad interest across oncology,^7,11^ including breast cancer.^12–14^

**Fig. 1:**
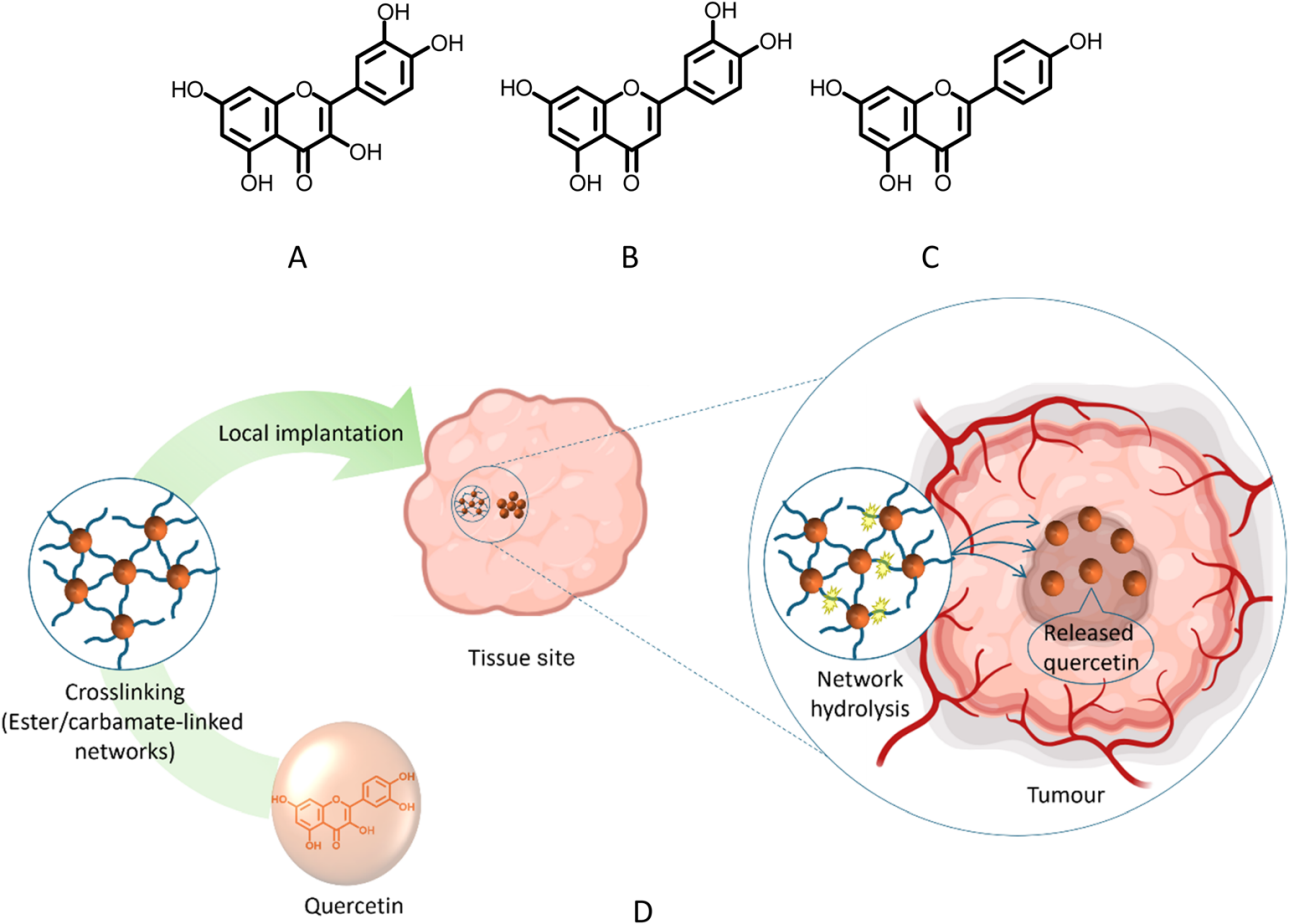
Structures of (A) quercetin (B) luteolin (C) apigenin (D) local extracellular depot concept in a breast cancer setting (a cross-linked flavonoid network placed adjacent to tumour tissue or within/around a resection cavity undergoes hydrolysis over time, enabling sustained release of flavonoid into the surrounding extracellular environment).

Pharmacokinetic challenges are particularly limiting for polyphenolic natural products such as flavonoids,^15^ which are widely reported to have anticancer activity.^15–17^ However, only a limited number of flavonoid-derived agents have progressed to clinical evaluation and, in some jurisdictions, regulatory approval (*e.g.*, icaritin/Ailading®), whereas others such as alvocidib/flavopiridol remain in clinical development.^18–20^ More broadly, flavonoids suffer from low aqueous solubility^21^ and extensive phase-II metabolism^22–24^ limiting sustained exposure following conventional administration.^15,21–24^ Quercetin, luteolin and apigenin are representative flavonoids (see Fig. 1A–C) with reported anti-proliferative effects in breast cancer cell models.^25,26^ Preclinical studies reported that quercetin inhibited MCF-7 cell viability with an IC_50_ of 13.7 ± 2.61 µM.^25^ Apigenin and luteolin also demonstrated concentration-dependent reductions in viability in MCF-7 and MDA-MB-231 cells, with IC_50_ values of 30 µM and 28 µM for apigenin and 43 µM and 27 µM for luteolin, respectively.^26^ Despite their promising therapeutic potentials, the clinical application of these flavonoids remains hindered by issues related to their bioavailability, metabolism and stability.^15,21–24^

To address the limitations of flavonoids in clinical applications, various formulation strategies have been explored to enhance apparent solubility, stability and delivery, including polymeric micelles,^27^ polymeric nanoparticles,^28^ liposomes,^29^ nanoemulsions,^30^ inorganic nanocarriers^31^ and dendritic systems.^32^ Representative examples include lecithin-stabilised polymeric micelles evaluated with pharmacokinetic and combination-therapy studies,^33^ pH-responsive polymeric nanoparticles and hybrid nanoparticle systems designed to modulate potency or resistance phenotypes,^34,35^ and liposomal or nanoemulsion formulations developed to improve handling and exposure^36,37^ While such approaches can improve formulation feasibility and *in vitro* performance, they can also be limited by practical translational constraints, including achievable drug loading,^38^ the need for stabilising excipients/surfactants,^39^ dose-scaling requirements,^40–42^ and variable *in vivo* behaviour.^43,44^ These considerations motivate alternative delivery formats in which the active agent is integrated into an insoluble depot matrix and release is governed by programmed degradation chemistry rather than formulation-dependent solubilisation.

Polymeric implantable devices offer a sustained, site-specific delivery strategy by maintaining therapeutic levels locally and they can be designed to gradually degrade, thereby supporting prolonged release without reliance on patient compliance.^7^ Degradation-controlled release of bioactives is a well-established design strategy in pioneering systems of biodegradable polymer-based delivery systems developed by Langer,^45–48^ Uhrich,^49–52^ Gillies^53,54^ and Adronov.^55,56^ These approaches employ hydrolysable linkages, responsive architectures and hydrophilic segments to tune degradation and release behaviour. A proof-of-concept implant formulation strategy reported by Rannard and co-workers, based on polymer prodrugs generated through the cross-linking of multifunctional drugs with alkyl chloroformates, demonstrated that this approach can yield sustained release implant materials with high drug loading and uniform distribution of the active within the insoluble network.^57^

In this study, inspired by prior sustained-release implant concepts based on multifunctional drugs used as structural components in cross-linked networks,^57^ (see Fig. 1D), we exploit the multiple hydroxyl groups of quercetin, luteolin and apigenin to construct insoluble cross-linked matrices *via* esterification or carbamate bond formation. Compared with the extensive literature on carrier-based flavonoid formulations, degradable cross-linked network depots that incorporate the flavonoid into the matrix architecture are less frequently reported.^27–32^ We therefore hypothesised that linker chemistry would govern hydrolytic degradation and, in turn, modulate release of intact flavonoid payloads under aqueous conditions relevant to local extracellular depot use, with breast cancer used here as the exemplar disease context. The resulting networks were synthesised and characterised, and their release behaviour and degradation were evaluated under controlled pH conditions with analytical verification of released species. Biological response was then assessed using extract-based exposure of MCF-7 cells benchmarked against free flavonoid standards, with additional cytocompatibility assessment in a non-malignant fibroblast model (161BR).

## Materials and methods

### Materials

Quercetin (98%), luteolin (97%), apigenin (95%) and 3,6,9-trioxaundecanedioic acid (95%) were purchased from Fluorochem Ltd (UK). [2-(2-Methoxyethoxy)ethoxy]acetic acid (95%) and hexamethylene diisocyanate (98%) were purchased from Tokyo Chemical Industry UK Ltd. Anhydrous pyridine was purchased from Merck (UK). Phosphate buffered saline (PBS) tablets were purchased from Fisher Scientific UK Ltd. All purchased materials were used as received. Dichloromethane was dried by using an MBRAUN SP7 solvent purification system fitted with activated alumina columns. The human breast cancer cell line MCF-7 (estrogen receptor-positive) and the human skin fibroblast cell line 161BR (European Collection of Authenticated Cell Cultures, ECACC) were obtained from Sigma-Aldrich (Gillingham, UK). Cells culture media Roswell Park Memorial Institute (RPMI 1640, Gibco™) and Minimum Essential Medium Eagle (MEME) were purchased from Fisher Scientific UK Ltd and supplemented with fetal bovine serum (FBS) from Fisher Scientific UK Ltd.

### Characterisation

Fourier-Transform Infrared (FT-IR) spectroscopic analyses were carried out over 4000–650 cm^−1^ at room temperature using a PerkinElmer 100 FT-IR instrument equipped with a diamond-ATR sampling accessory. The resulting spectra were analysed using the PerkinElmer spectrum IR software (version 10.6.2). High Performance Liquid Chromatography (HPLC) analysis of the flavonoids in the *in vitro* release study was performed using an Agilent-1100 series HPLC equipped with a reverse phase column: C-18 (ACE-221-2546; 4.6 mm × 250 mm; 5 µm). Ultraviolet absorbances of the flavonoids within side-by-side cell diffusion study were recorded using a UV-vis spectrophotometer V-530 (Jasco, UK) over the range 200–350 nm. Mass spectrometry (MS) was conducted using a Thermo Fisher Scientific Orbitrap XL LCMS. The sample was introduced by liquid chromatography (LC) and sample ionization was achieved by electrospray ionization (ESI). Differential scanning calorimetry (DSC) measurements were performed on a TA Instruments DSC Q2000 adapted with a TA Refrigerated Cooling System (RCS90), using aluminium TA Tzero pans and lids, measuring from −80 °C to 100 °C with heating and cooling rates of 5 °C min^−1^ under nitrogen gas with a flow rate of 50 mL min^−1^. Thermogravimetric analysis (TGA) was carried out on TA Instruments TGA Q50 instrument with aluminium Tzero pans. The sample was heated from 20 °C to 600 °C at 10 °C min^−1^ under nitrogen gas with a flow rate of 60 mL min^−1^. Thermal characterisation of samples was investigated using the TA Instruments Universal Analysis 2000 software (version 4.5A).

### General method for the synthesis of cross-linked esters 1–3

3,6,9-Trioxaundecanedioic acid (2.0 g, 9.0 mmol, 1.0 eq.) was dissolved in dry dichloromethane (40 mL) and cooled in an ice-bath under argon. Oxalyl chloride (3.4 g, 27.0 mmol, 3.0 eq.) was added dropwise to the solution with a catalytic amount of *N*,*N-*dimethylformamide (1 drop). The mixture was stirred for one hour, still in an ice bath. The mixture was then removed from the ice bath and stirred for a further 24 hours at room temperature. The solvent and excess of oxalyl chloride were removed under reduced pressure. The produced acyl chloride was re-dissolved in dry dichloromethane (40 mL) under argon in an ice bath. To this stirred solution, flavonoid in anhydrous pyridine (5–7 mL) was added dropwise over the course of one hour. The mixture was then removed from the ice bath and stirred for a further 24 hours at room temperature. The product was filtered and washed with tetrahydrofuran (100 mL), and acetone (75 mL) then dried under vacuum.

Each cross-linked polyester derivative was synthesised using the same method but with the following specific conditions:

Cross-linked 1: quercetin (388.3 mg, 1.2 mmol) in anhydrous pyridine (5 mL). mp. 230–233 °C. IR *v*_max_/cm^−1^ = 3085 (C–H arene), 2917 (C–H), 1779 (C=O ester), 1650 (C=O ketonic carbonyl), 1622 (C=C) 1503, 1478, 1464, 1428 (ring mode) 1097 (asymm. O–C–O) (see Fig. S1).

Cross-linked 2: luteolin (429.0 mg, 1.5 mmol) in anhydrous pyridine (5 mL). mp. 191–192 °C. IR *v*_max_/cm^−1^ = 3085 (C–H arene), 2917 (C–H), 1735 (C=O ester), 1631 (C=O ketonic carbonyl),1609 (C=C) 1576, 1503, 1451, 1427 (ring mode) 1094 (*asymm*. O–C–O) (see Fig. S2).

Cross-linked 3: apigenin (486.0 mg, 1.8 mmol) in anhydrous pyridine (7 mL). mp. 135–138 °C. IR *v*_max_/cm^−1^ = 3066 (C–H arene), 2913 (C–H), 1770 (C=O ester), 1633 (C=O ketonic carbonyl),1615 (C=C), 1539, 1505, 1486, 1417 (ring mode) 1098 (asymm. O–C–O) (see Fig. S3).

### General method for the synthesis of cross-linked carbamates 4–6

Hexamethylene diisocyanate (1.4 g, 8.2 mmol, 2.5 eq.) was dissolved in dry dichloromethane (40 mL) and cooled in an ice-bath under argon. The flavonoid (1.0 eq.) in anhydrous pyridine (5–7 mL) was added dropwise over the course of one hour. The mixture was then removed from the ice bath and stirred for a further 72 hours at room temperature. The reaction mixture was then diluted with dichloromethane (30 mL) followed by precipitation dropwise in cold hexane (1 L) with stirring. The cross-linked product was filtered and washed with tetrahydrofuran (50 mL), and acetone (100 mL) then dried under vacuum.

Each cross-linked polycarbamate was synthesised using same method but with the following specific conditions:

Cross-linked 4: quercetin (1.0 g, 3.3 mmol) in anhydrous pyridine (5 mL). mp. 240–242 °C. IR *v*_max_/cm^−1^ = 3318 (N–H), 3038 (C–H arene), 2929 (C–H), 1721 (C=O carbamate), 1651 (C=O ketonic carbonyl), 1615 (C=C), 1572, 1532, 1495, 1462 (ring mode) (see Fig. S4).

Cross-linked 5: luteolin (1.0 g, 3.4 mmol) in anhydrous pyridine (5 mL). mp. 225–228 °C. IR *v*_max_/cm^−1^ = 3320 (N–H), 3036 (C–H arene), 2929 (C–H), 1719 (C=O carbamate), 1653 (C=O ketonic carbonyl), 1618 (C=C), 1570, 1538, 1494, 1432 (ring mode) (see Fig. S5).

Cross-linked 6: apigenin (1.0 g, 3.7 mmol) in anhydrous pyridine (7 mL). mp. 185–186 °C. IR *v*_max_/cm^−1^ = 3290 (N–H), 3074 (C–H arene), 2929 (C–H), 1726 (C=O carbamate), 1651 (C=O ketonic carbonyl), 1604 (C=C), 1574, 1556, 1494, 1441 (ring mode) (see Fig. S6).

### *In vitro* flavonoid release study

For each 1 mg sample of cross-linked derivative, phosphate-buffered saline (PBS; 10 mL, pH 7.4, 6.5 or 5.0) was added. Samples were incubated at 37 °C with shaking (160 rpm). At predetermined time points (0, 2, 4, 8, 12, 24, 48, 72, 144 and 288 h), aliquots were withdrawn for HPLC-UV analysis of released flavonoid. Quantification was performed using external calibration (see Fig. S7–S9) with authentic flavonoid standards (linear over the working range; *R*^2^ > 0.99). Results are reported as mean ± SEM from triplicate experiments.

### Determination of flavonoid content (wt/wt%) by exhaustive degradation

Drug loading (wt/wt%) was determined by exhaustive degradation of the cross-linked networks under acidic methanolic–aqueous conditions selected to enable complete release while minimising degradation of the parent flavonoids. Networks (5 mg) were suspended in 30 mL of methanolic–aqueous acidic medium (pH 2.0) and incubated at 60 °C with stirring (400 rpm). Aliquots were withdrawn at 0, 12, 24 and 48 h and analysed by HPLC-UV using external calibration with authentic flavonoid standards prepared in the same medium (linear over the working range; *R*^2^ > 0.99). A plateau in released flavonoid was observed by 12 h, with 24 and 48 h confirming no further increase (see Fig. S10). To confirm stability of the parent flavonoids under these conditions, quercetin, luteolin and apigenin standards were incubated in parallel under identical conditions and analysed by HPLC-UV, showing no evidence of additional peaks attributable to degradation and no meaningful loss of the parent peak over the experiment timeframe (see Fig. S10). Drug loading (wt/wt%) was calculated from the total mass of flavonoid recovered at the plateau relative to the initial dry network mass. Results are reported as mean ± SEM from triplicate experiments.

### Side-by-side diffusion procedure (horizontal diffusion cells)

Side-by-side diffusion studies were conducted using horizontal side-by-side diffusion cells with an effective diffusion area of 1.76 cm^2^ and a chamber volume of 5 mL. A synthetic cellulose membrane (MW cut-off 1000 Da) was positioned vertically between the donor and receiver chambers. Prior to assembly, membranes were hydrated by pre-soaking in PBS (pH 5.0, 6.5 or 7.4) at 37 °C for 30 min. Both chambers were maintained at 37 ± 0.5 °C with continuous stirring (160 rpm) to minimise concentration gradients.

Cross-linked networks (1 mg) were placed in the donor chamber and PBS (5 mL; pH 7.4, 6.5 or 5.0) was added. At defined time points (2, 4, 8, 12, 24, 48, 72, 96, 144, 240 and 360 h), 500 µL aliquots were withdrawn from both donor and receiver chambers for analysis and returned to the corresponding chamber to maintain constant volume. Results are reported as mean ± SEM from triplicate experiments.

### Biological assays: cell culture

#### Preparation of test samples

Cross-linked networks were sanitised by ethanol (0.5 mL) and allowed to evaporate overnight under sterile conditions. As the cross-linked networks are insoluble, liquid extracts of the samples were prepared by exposing each 1 mg cross-linked flavonoid material to complete the relevant cell culture medium (10 mL) at 37 °C for either 5 min or 24 h, to assess whether biologically active species leach out into the medium. Extracts were then filtered through a 0.2 µm syringe filter and applied to the cells.^58^ For MCF-7, complete medium comprised RPMI 1640 supplemented with 5% FBS and 1% antibiotic/antimycotic solution (penicillin/streptomycin/amphotericin B); extracts were applied neat (100%) or diluted (75%, 50%, 25%, 10%, 5% and 1%). For 161BR, complete medium comprised minimum essential medium eagle (MEME) supplemented with 2 mM glutamine, 15% FBS, 1% non-essential amino acids and 1% antibiotic/antimycotic solution (penicillin/streptomycin/amphotericin B); extracts were applied only neat (100%) for the 5 minute and 24 hour conditions.

#### Cell viability assay (MTT)

MCF-7 and 161BR cells were seeded at a density of 4 × 10^4^ cells per mL in 96-well plates and incubated for 24 hours to allow cell attachment. MCF-7 cells were then treated with serial dilutions (100%, 75%, 50%, 25%, 10%, and 1%) of the network extracts (prepared as described above), alongside positive control treatments using free flavonoid solutions at 50, 25, 10, and 1 µM, and a negative control medium, while 161BR cells were treated with neat (100%) network extracts only (prepared as described above), alongside a positive control treatment using a free flavonoid solution (50 µM) and a negative control medium. Following a 67 hour incubation, 20 µL of MTT reagent (5 mg mL^−1^ in PBS) was added to each well and the plates were incubated for an additional 5 hours at 37 °C (thus arriving at 72 h). After incubation with the MTT solution, the medium was removed, and the resulting purple formazan crystals were solubilised by adding 100 µL of DMSO to each well. Absorbance was measured at 570 nm using a SpectraMax UV microplate reader. Each condition was tested in triplicate across three independent experiments. Viability was calculated as a percentage relative to untreated control cells, which were set at 100% viability. Cell viability data are expressed as mean ± standard error of the mean (SEM).

#### Statistical analysis

Data are presented as mean ± standard error of the mean (SEM). Statistical analysis of free flavonoid controls and network extracts from cross-linked networks 1–6 in MCF-7 and 161BR cells was performed using two-way ANOVA with Bonferroni *post hoc* testing. Statistical significance was set at *p < 0.05* (specifically, *p < 0.05, **p < 0.01, ***p < 0.001).

## Results and discussion

### Synthesis of cross-linked flavonoid networks

This investigation explored the cross-linking of flavonoids to create biodegradable drug delivery materials that may be suitable for deployment as implants, by exploiting the reactive hydroxyl groups of quercetin, apigenin, and luteolin to form cleavable cross-links. It is well-established that phenolic esters^56^ and phenolic carbamates^59^ are susceptible to cleavage under aqueous conditions, with enzymatic processes potentially providing a complementary release pathway *in vivo*.^60^ This cleavable property is vital for ensuring sustained release of the therapeutic flavonoid payloads^61^ from a cross-linked network upon hydrolytic degradation.

**Scheme 1:**
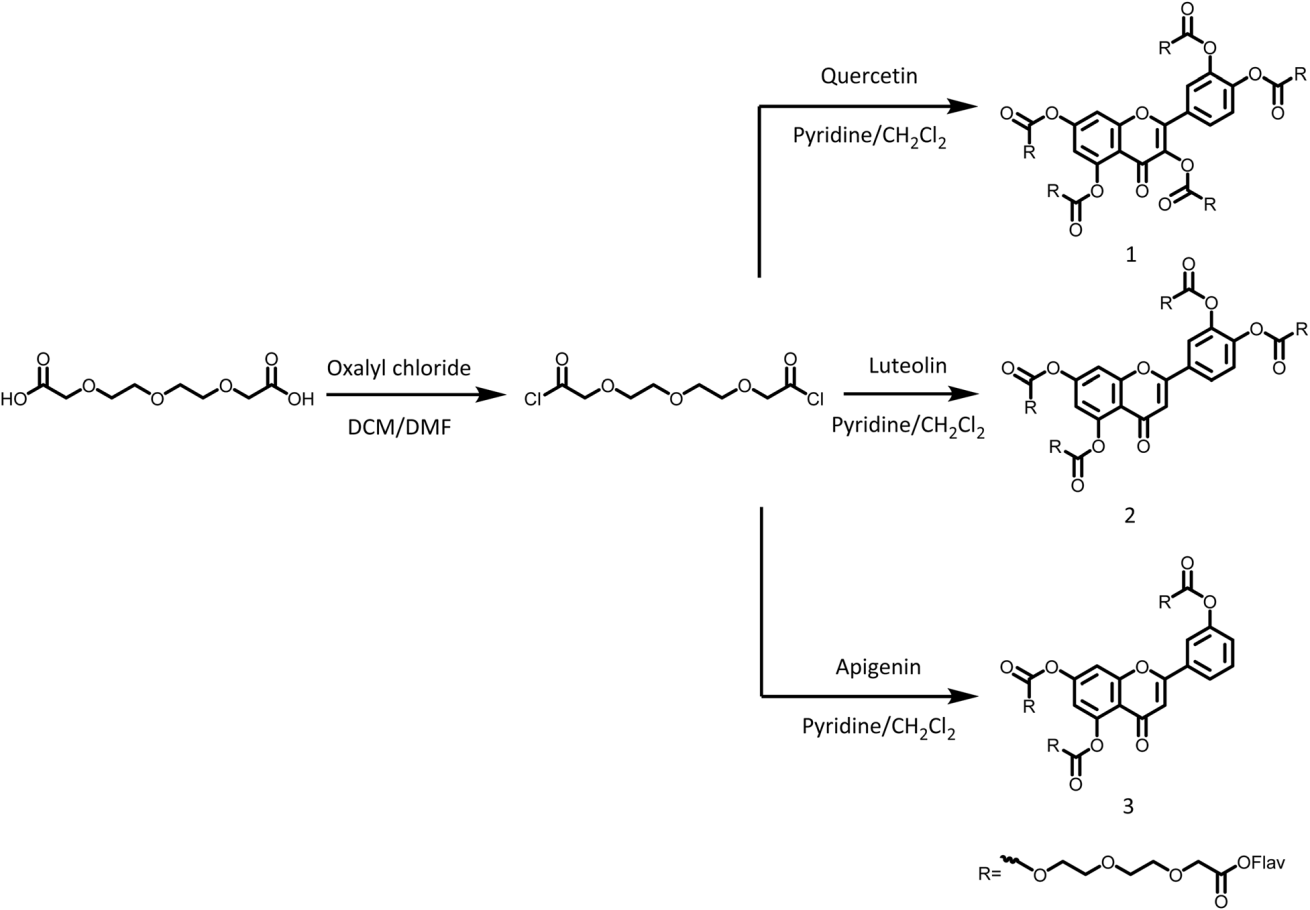
Synthetic strategy for cross-linked flavonoid derivatives 1, 2 and 3 that contained ester linkages.

**Scheme 2:**
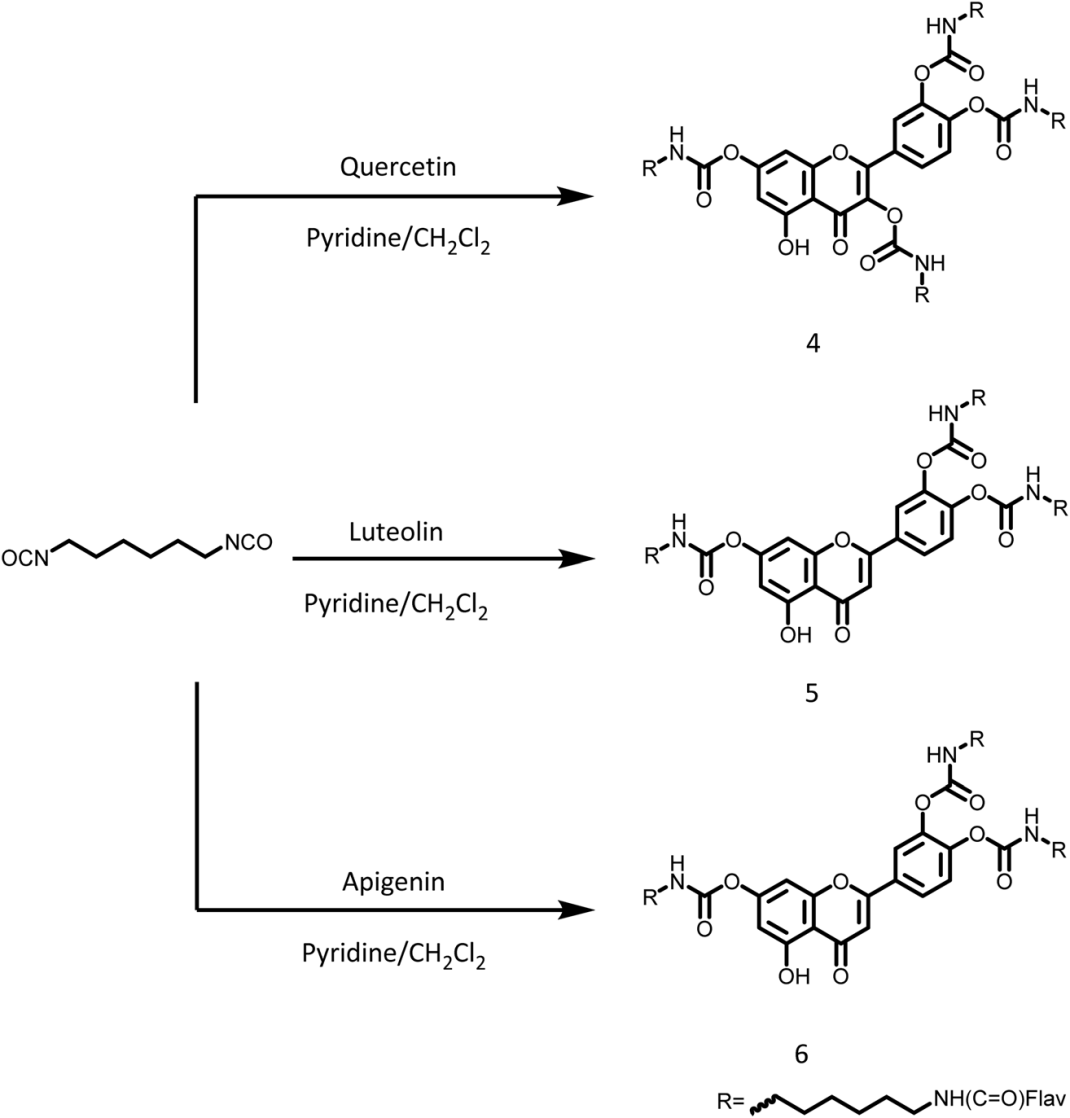
Synthetic strategy for cross-linked flavonoid derivatives 4, 5 and 6 that contained carbamate linkages.

Cross-linked network systems (1–6) were therefore prepared to enable controlled drug release of these flavonoids under aqueous conditions relevant to local depot use. Two distinct approaches to the cross-linked drug delivery systems were adopted. Ester-linked networks (1–3) and carbamate-linked networks (4–6) were prepared by reacting the multifunctional hydroxyl groups of these flavonoids with either 3,6,9-trioxaundecanedioyl chloride (Scheme 1) or hexamethylene diisocyanate (Scheme 2), respectively. The inherent multifunctionality of flavonoids, and bifunctional cross-linkers facilitated the creation of the cross-linked network architectures.

### Characterisation of cross-linked flavonoid networks

Network formation was confirmed by FTIR spectroscopy. In the case of the esters 1–3, strong carbonyl absorptions were observed at 1779, 1735 and 1770 cm^−1^, respectively, corresponding to the ester carbonyl (C=O) stretching vibrations (Fig. S1–S3). For the carbamate-linked networks 4–6 (see Scheme 2), carbonyl bands were observed at 1721, 1719 and 1726 cm^−1^ together with N–H stretching bands at 3318, 3320 and 3290 cm^−1^, respectively, corresponding to carbamate C=O and N–H stretching vibrations (Fig. S4–S6).

The thermal stabilities of the solid cross-linked compounds were evaluated using thermogravimetric analysis (TGA) and differential scanning calorimetry (DSC). The TGA data (Fig. S11–S14) showed the onset of mass loss at 104 °C for the apigenin-derived ester network 3 and at 220 °C for the quercetin-derived ester network 1. The quercetin-derived carbamate network 4 exhibited an earlier onset of decomposition (198 °C) than its ester-linked counterpart 1, indicating that thermal stability in this series depends on both linkage chemistry and flavonoid scaffold. DSC analysis between −80 and 100 °C (Fig. S15–S18) did not show a melting transition attributable to crystalline melting; only broad thermal features were observed within this range. Overall, the DSC and TGA results indicate that the networks maintain appropriate thermal stability and remain as solid materials without major phase changes across sub-ambient to physiological temperatures.

### *In vitro* drug release and degradation analysis

**Fig. 2:**
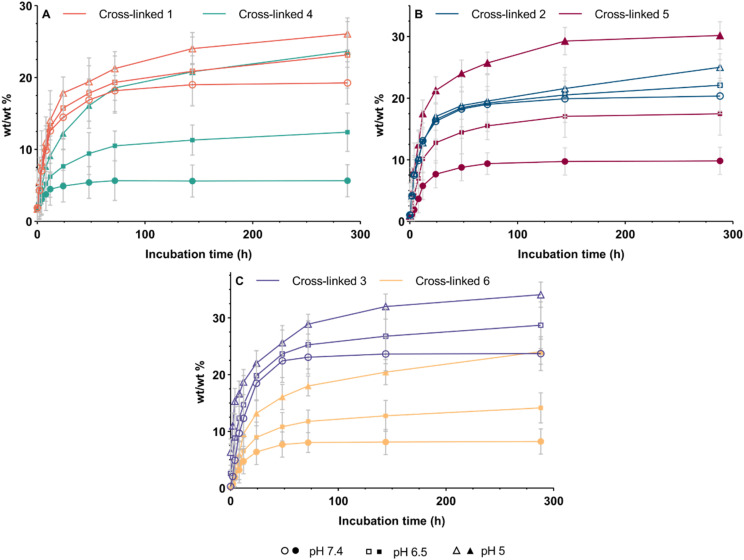
Flavonoid release profiles (*in vitro*) from cross-linked polymer networks over 288 hours (A) 1 and 4; (B) 2 and 5; (C) 3 and 6, with samples incubated in PBS at pH 5.0, 6.5 or 7.4. Data are expressed as mean ± SEM. (*n* = 3).

**Fig. 3:**
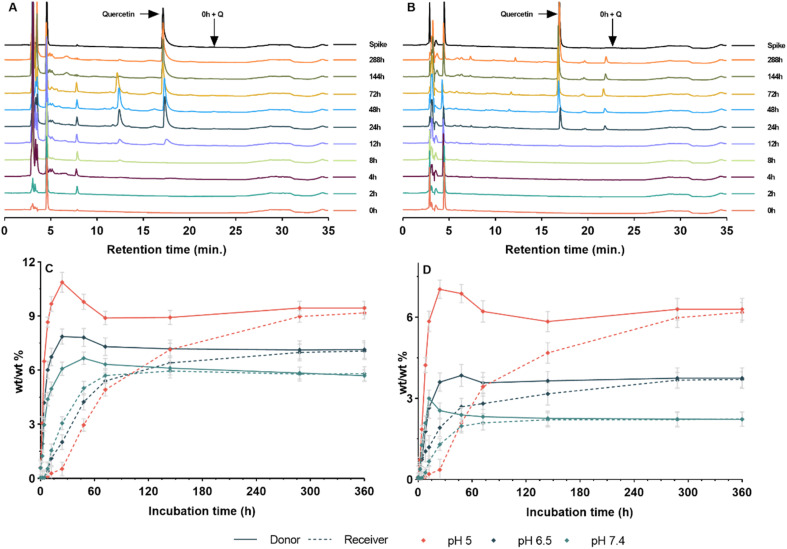
(A and B) HPLC chromatograms of quercetin release (*in vitro*) over 288 hours from cross-linked product 1 incubated in PBS at pH 7.4 (A) and pH 5 (B). (C and D) Diffusion profiles of apigenin release (*in vitro*) over 360 hours from the donor to the receiver chamber of the diffusion system from cross-linked products 3 (C) and 6 (D) in pH 7.4 and pH 5. Data are expressed as mean ± SEM. (*n* = 3).

The central hypothesis of this study was that hydrolysable linkages embedded within the cross-linked flavonoid networks would enable time-dependent degradation and controlled release of intact flavonoid payloads, and that the rate of release could be mediated based on linker chemistry (ester *vs.* carbamate) and the flavonoid scaffolds. To quantify release, networks 1–6 were incubated in PBS at 37 °C under three pH conditions (7.4, 6.5 and 5.0) (see Fig. 2, 3, and S19); pH 7.4 was used as a physiological extracellular benchmark, while pH 6.5 was selected as a mildly acidic condition representative of tumour extracellular pH, which is typically weakly acidic in bulk (commonly 6.0–7.0).^62,63^ pH 5.0 was included as a stringent acidic benchmark to probe pH-responsiveness under more strongly acidic conditions, consistent with reports that localised extracellular acidity at the cancer cell surface can be substantially lower than bulk tumour extracellular pH (*e.g.* <5.5).^63,64^

Aliquots were analysed by HPLC using external calibration (*R*^2^ > 0.99) with standard retention times and spiking controls to confirm parent-flavonoid identity (see Fig. 3A and B). Release is reported as cumulative release (wt/wt% relative to network mass). Total flavonoid content of each network was determined under forced-degradation conditions (pH 2, 60 °C, stirring), giving loading values of 28, 34 and 36 wt/wt% for ester networks 1–3, and 33, 39 and 44 wt/wt% for carbamate networks 4–6, respectively.

Across the ester-linked series (1–3), cumulative release increased with decreasing pH (see Fig. 2A–C). By 288 h, cross-linked 1 released 19 (pH 7.4), 23 (pH 6.5) and 26 wt/wt% (pH 5.0), cross-linked 2 released 21, 22 and 25 wt/wt%, and cross-linked 3 released 24, 29 and 34 wt/wt%, respectively. Relative to the experimentally measured loadings (28, 34 and 36 wt/wt% for cross-linked networks 1–3), the pH 5.0 condition approached near-complete release for networks 1 and 3, whereas network 2 released a smaller fraction of its measured loading after a period of 288 h.

In contrast, the carbamate-linked series (4–6) exhibited markedly stronger pH-responsiveness with substantially lower release at pH 7.4 but pronounced increases under acidic conditions. By 288 h, cross-linked 4 released 6 (pH 7.4), 12 (pH 6.5) and 24 wt/wt% (pH 5.0), cross-linked 5 released 10, 18 and 30 wt/wt%, and cross-linked 6 released 8, 14 and 24 wt/wt%, respectively. When benchmarked against measured loading (32, 39 and 44 wt/wt% for networks 4–6), cross-linked 6 showed the lowest fractional release at pH 5.0, while the cross-linked network 5 demonstrated the highest acidic release within the carbamate series (see Fig. 2A–C).

Side-by-side diffusion studies complemented the bulk release data by confirming that the released species accumulating in the receiver compartment were able to cross the 1 kDa membrane,^65^ consistent with the release of low-molecular-weight species rather than bulk network material (see Fig. 3 and S20). Receiver-chamber accumulation followed the same trend across pH as the bulk release profiles (pH 7.4 < 6.5 < 5.0). Notably, the apigenin network 3 exhibited the highest receiver-chamber accumulation under acidic conditions (receiver 10 wt/wt% at pH 5.0, 360 h), whereas the corresponding carbamate network 6 delivered a lower amount to the receiver (7 wt/wt% at pH 5.0, 360 h) (see Fig. 3). Together with retention-time matching and spiking controls, these data are consistent with release of the parent flavonoid payload.

Overall, these results show that linker chemistry is a primary determinant of pH-dependent release, with carbamate networks showing the greatest separation between pH 7.4 and acidic conditions, while ester networks provide comparatively higher baseline release at pH 7.4 and smaller pH-driven shifts. The pH 6.5 condition, selected to reflect mildly acidic tumour extracellular pH, showed intermediate release behaviour across the series. These data support the proposed hypothesis, demonstrating tuneable, time-dependent release from the cross-linked networks over extended incubation periods, controlled by linker chemistry, pH and flavonoid scaffold.

### Cytotoxicity of flavonoid networks

**Fig. 4:**
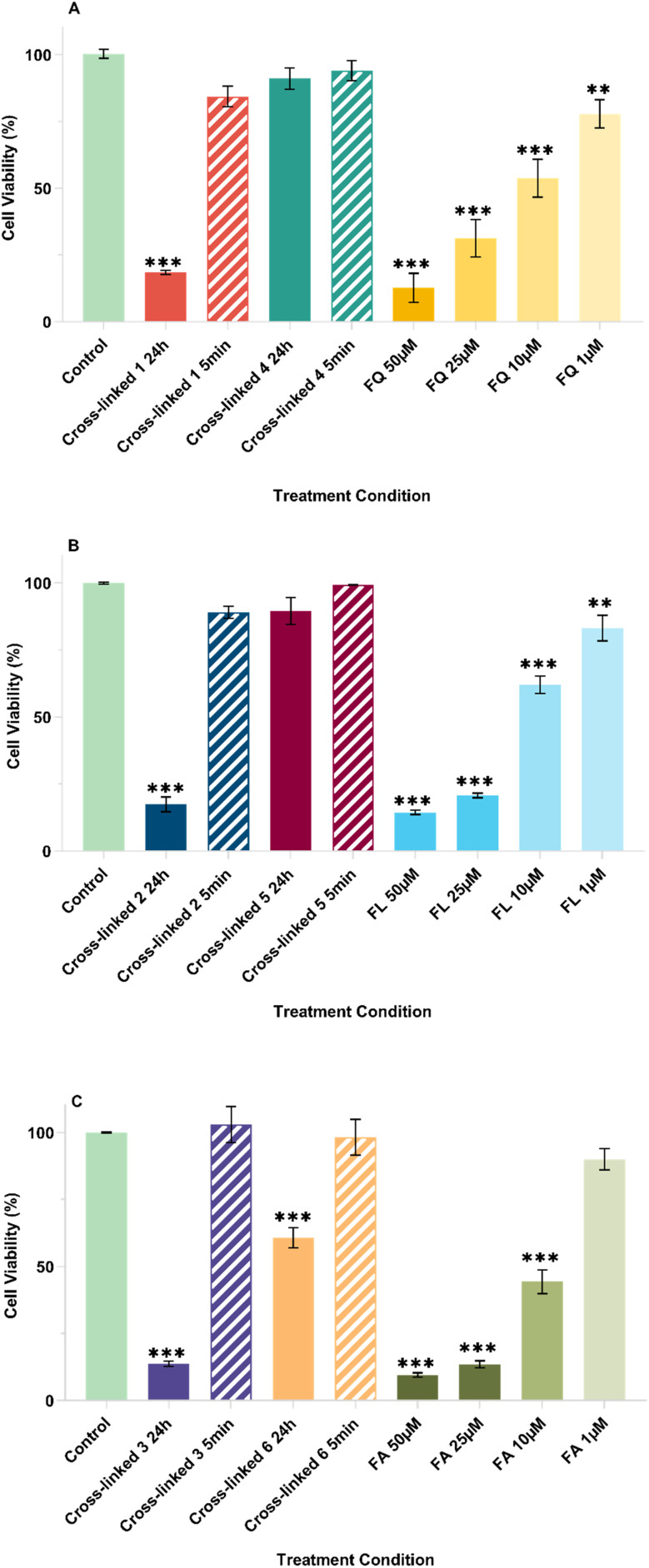
Anti-proliferative activities of undiluted (100%) liquid extracts from cross-linked networks, compared to several concentrations (1–50 µM) of free flavonoids, against MCF-7 cell line using an MTT assay. (A) Cross-linked 1, 4 and free quercetin (FQ); (B) cross-linked 2, 5 and free luteolin (FL); C) Cross-linked 3, 6 and free apigenin (FA). Cell culture medium was pre-exposed to the cross-linked networks for 24 hours (solid bars) or 5 minutes (striped bars) prior to application. Data are expressed as mean ± SEM (*n* = 3).

To assess whether antiproliferative activity is retained following release from the cross-linked networks, an extract-based MTT viability assay was performed using MCF-7 breast cancer cells (see Fig. 4 and S21–S23) and the non-malignant fibroblast line 161BR (see Fig. S24). In both cases, cells were treated with culture medium that had been pre-exposed to the cross-linked materials for defined times (24 h to allow release into the medium, and 5 min as a short-exposure control to probe rapidly leachable species). Cell viability was quantified after 72 h and compared with untreated controls.

For MCF-7, the 24 h pre-exposure results revealed a clear correspondence between linkage chemistry, release behaviour and antiproliferative activity. The ester-linked quercetin network (cross-linked 1) reduced viability to 19%, significantly below the control (*p* < 0.001; Fig. 4A), whereas the carbamate network 4 showed no cytotoxicity after 24 h pre-exposure (91%, *p* > 0.05 *vs.* control). Under the short pre-exposure condition (5 min), neither network reduced viability (84% for cross-linked 1 and 94% for cross-linked 4; *p* > 0.05 *vs.* control), supporting that the cytotoxicity observed after 24 h pre-exposure is consistent with time-dependent release rather than rapid leaching (Fig. 4A and S21A–E). The activity of the 24 h extract from cross-linked 1 was benchmarked against free quercetin standards tested in the same assay (*e.g.*, 31% at 25 µM and 13% at 50 µM; *p* > 0.05 *vs.*1 in both cases).

Cytotoxicity results for the luteolin and apigenin networks followed the same pattern. After 24 h pre-exposure, the ester-linked networks reduced viability to 17% (cross-linked 2) and 14% (cross-linked 3) (*p* < 0.001 *vs.* control), whereas the corresponding carbamate networks showed substantially higher viability (cross-linked 5 : 90%, cross-linked 6 : 61%; see Fig. 4B, C and S22–S23). Under the 5 min pre-exposure condition, none of the networks (2, 5, 3, 6) differed significantly from the negative control (*p* > 0.05).

Cytocompatibility testing in the non-malignant fibroblast line 161BR showed no cytotoxicity under the matched extract conditions (see Fig. S24). For all networks and both exposure conditions, viability remained above 90% and was not significantly different from the untreated control (*p* > 0.05).

Taken together, these results show that the flavonoid-derived cross-linked networks were successfully formed as insoluble matrices with high flavonoid loading and tunable release behaviour. Release of intact parent flavonoids was governed primarily by linker chemistry and pH. Ester-linked networks showed higher release than the corresponding carbamate-linked networks at pH 7.4, while release increased as pH decreased across both linker classes, with the carbamate series showing more pronounced pH dependence. The biological data broadly reflected these release patterns, with linker-dependent differences in antiproliferative activity that were most evident in the 24 h MCF-7 extract experiments. In parallel, the matched 161BR extract conditions remained non-cytotoxic, supporting cytocompatibility under the conditions tested. Collectively, these findings support degradable cross-linked flavonoid networks as a promising platform for tunable local depot delivery.

## Conclusion

In this study, quercetin, luteolin and apigenin were used as multifunctional structural components to prepare insoluble cross-linked flavonoid networks with either ester (1–3) or carbamate (4–6) linkages, as degradable matrices intended for sustained local extracellular delivery. Quantification of total flavonoid content by exhaustive degradation established high loadings across all networks, enabling interpretation of release as a fraction of payload rather than concentration alone. Release studies in PBS at 37 °C across pH 7.4, 6.5 and 5.0 demonstrated that linker chemistry is a primary determinant of pH-dependent behaviour: ester networks provided higher baseline release at pH 7.4 with smaller pH-driven shifts, whereas carbamate networks exhibited low release at pH 7.4 but pronounced increases under acidic conditions. Across the network series, release at pH 6.5 was consistently intermediate between that observed at pH 7.4 and pH 5.0. Side-by-side diffusion experiments further supported release of low-molecular-weight species rather than bulk material. In MCF-7 cells, 24 h extracts from ester networks produced strong antiproliferative effects, while 5 min extracts were not cytotoxic, supporting time-dependent release as the dominant driver of biological response. In the non-malignant fibroblast line 161BR, viability after treatment with all extracts was non-significantly different from the untreated control, indicating no detectable cytotoxicity under the conditions tested. Taken together, these findings show that embedding flavonoids into degradable cross-linked architectures provides a route to tuneable, sustained release controlled by linker chemistry, pH and flavonoid scaffold.

## Author contributions

All of the authors conceived the work and designed the experiments; M. S. H. A. performed the experiments and processed the data; W. H., H. M. I. O. and F. G. supervised the project; All authors drafted and reviewed the manuscript.

## Conflicts of interest

The authors declare no competing financial interest.

## Supplementary Material

RA-016-D6RA02770A-s001

## Data Availability

The data supporting this article have been included as part of the supplementary information (SI). Supplementary information: GPC, TGA, DSC, MS, FTIR spectra, drug release profiles, and MTT assay graphs (Fig. S1–S24). See DOI: https://doi.org/10.1039/d6ra02770a.
