# Supplementary material for: Biodegradable cross-linked flavone networks featuring either ester or carbamate linkages for controlled delivery of anti-cancer agents
Source: RSC Adv. 2026 May 12;16(27):24986–96. doi: 10.1039/d6ra02770a (PMC13162338; doi:10.1039/d6ra02770a)
Supplement: RA-016-D6RA02770A-s001 [file RA-016-D6RA02770A-s001.pdf]

Supplementary Information (SI) for RSC Advances.
This journal is © The Royal Society of Chemistry 2026

Supporting Information

Biodegradable cross-linked flavone networks featuring either ester or carbamate linkages for controlled delivery of anti-cancer agents

Mohammed S.H. Alwaezi,[a,b] Francesca Greco,[c] Helen M.I. Osborn, *[c] and Wayne Hayes*[a]

[a]Department of Chemistry, University of Reading, Whiteknights, Reading RG6 6AD, United Kingdom; w.c.hayes@reading.ac.uk

[b]Department of Chemistry, Faculty of Science, University of Zakho, Zakho, 42002, Iraq.

[c]School of Pharmacy, University of Reading, Whiteknights, Reading RG6 6AD, United Kingdom; h.m.i.osborn@reading.ac.uk

Contents

Figure S1 IR spectrum of cross-linked **1**. 5
Figure S2 IR spectrum of cross-linked **2**. 6
Figure S3 IR spectrum of cross-linked **3**. 6
Figure S4 IR spectrum of cross-linked **4**. 7
Figure S5 IR spectrum of cross-linked **5**. 7
Figure S6 IR spectrum of cross-linked **6**. 8
Figure S7 Quercetin standard concentration curve for release studies. 8
Figure S8 Luteolin standard concentration curve for release studies. 9
Figure S9 Apigenin standard concentration curve for release studies. 9
Figure S10 Determination of flavonoid content (wt/wt%) by exhaustive degradation and stability of the parent flavonoids under these conditions 10
Figure S11 TGA thermogram of cross-linked **1**. 11
Figure S12 TGA thermogram of cross-linked **2**. 11
Figure S13 TGA thermogram of cross-linked **3**. 12
Figure S14 TGA thermogram of cross-linked **4**. 12

Figure S15 DSC thermogram of cross-linked **1**...13

Figure S16 DSC thermogram of cross-linked **2**...13

Figure S017 DSC thermogram of cross-linked **3**...14

Figure S18 DSC thermogram of cross-linked **4**...14

Figure S19 HPLC chromatograms of quercetin, luteolin and apigenin release (in vitro) over 288 hours from cross-linked products **2,3,4,5,** and **6** incubated in PBS 7.4 or 5. 16

Figure S20 Diffusion profiles of quercetin and luteolin release (in vitro) over 360 hours from the donor to the receiver chamber of the diffusion system from cross-linked products **1** and **4** respectively in pH 7.4 and pH 5. Data are expressed as mean ±SEM. (n=3) 17

Figure S21 Anti-proliferative activities of a series dilutions releasing medium from cross-linked **1** incubated for A) 24 hours and B) 5 minutes and cross-linked **4** incubated for C) 24 hours (solid bars) and D) 5 minutes (striped bars) compared to E) free quercetin standards, against MCF-7 cell line using an MTT assay. Data are expressed as mean ± SEM (n = 3). 19

Figure S22 Anti-proliferative activities of a series dilutions releasing medium from cross-linked **2** incubated for A) 24 hours and B) 5 minutes and cross-linked **5** incubated for C) 24 hours (solid bars) and D) 5 minutes (striped bars) compared to E) free luteolin standards, against MCF-7 cell line using an MTT assay. Data are expressed as mean ± SEM (n = 3). 21

Figure S23 Anti-proliferative activities of a series dilutions releasing medium from cross-linked **3** incubated for A) 24 hours and B) 5 minutes and cross-linked **6** incubated for C) 24 hours (solid bars) and D) 5 minutes (striped bars) compared to E) free apigenin standards, against MCF-7 cell line using an MTT assay. Data are expressed as mean ± SEM (n = 3). 23

Figure S24 Anti-proliferative activities of undiluted (100%) liquid extracts from cross-linked networks, compared to 50 µM of free flavonoids, against 161BR cell line using an MTT assay. A) Cross-linked 1, 4 and free quercetin (FQ); B) Cross-linked 2, 5 and free luteolin (FL); C) Cross-linked 3, 6 and free apigenin (FA). Cell culture medium was pre-exposed to the cross-linked networks for 24 hours (solid bars) or 5 minutes (striped bars) prior to application. Data are expressed as mean ± SEM (n = 3). 25

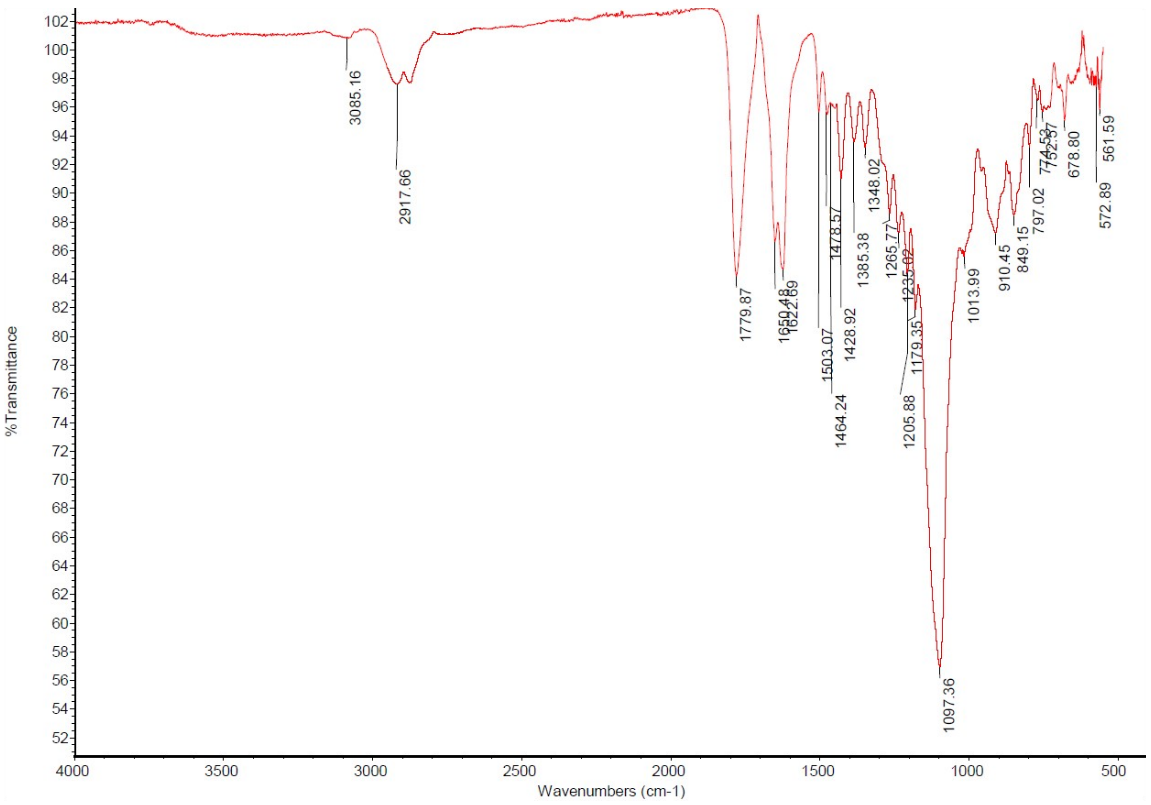

*Figure S1 IR spectrum of cross-linked **1**.*

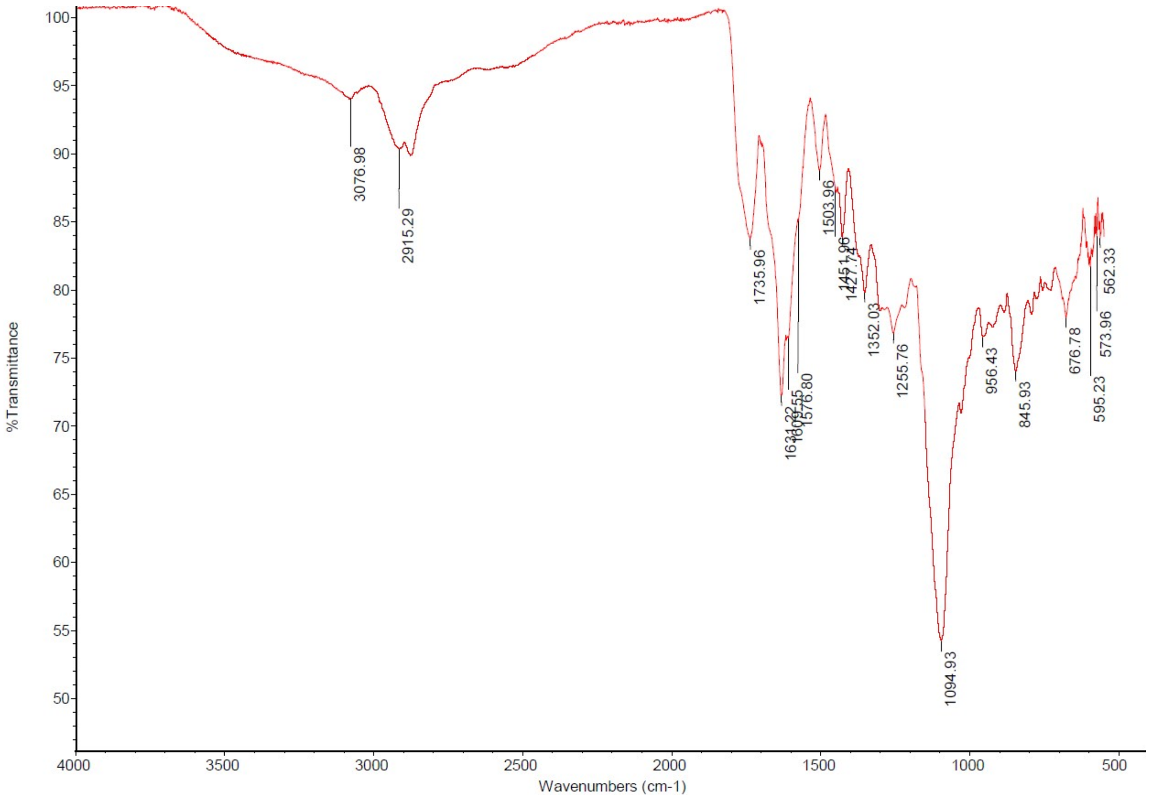

*Figure S2 IR spectrum of cross-linked **2**.*

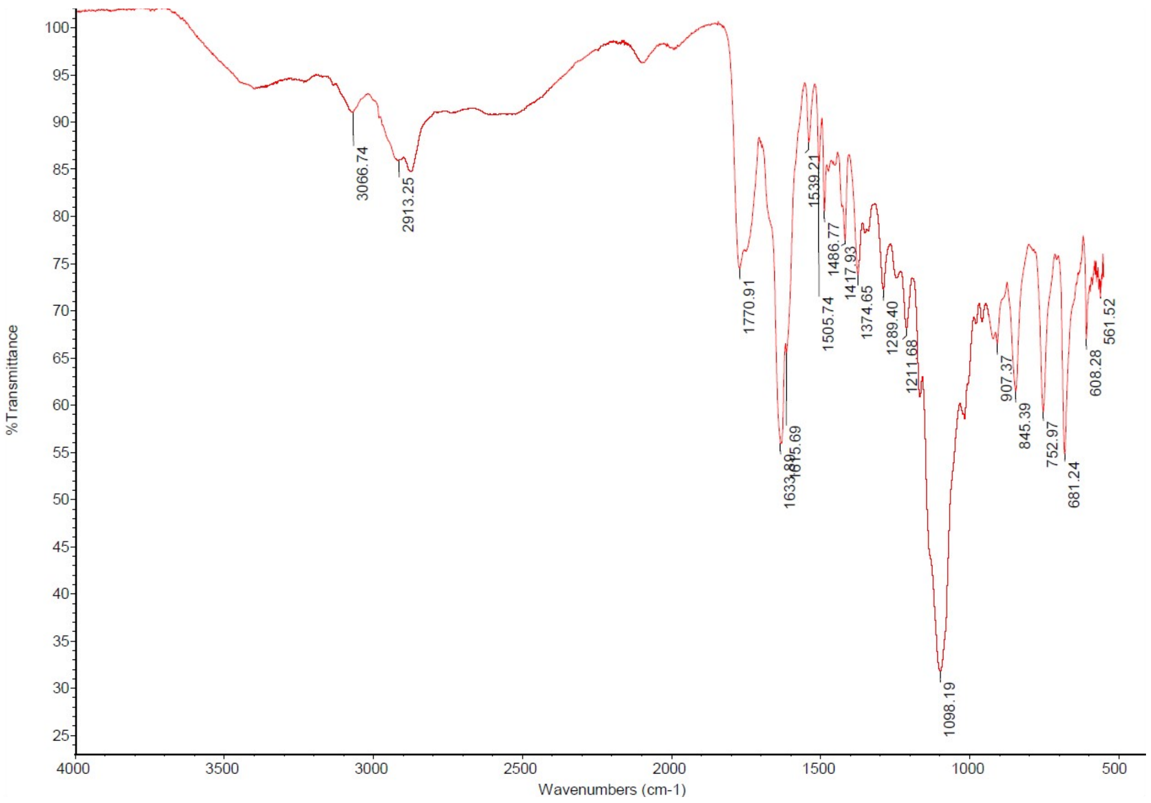

*Figure S3 IR spectrum of cross-linked **3**.*

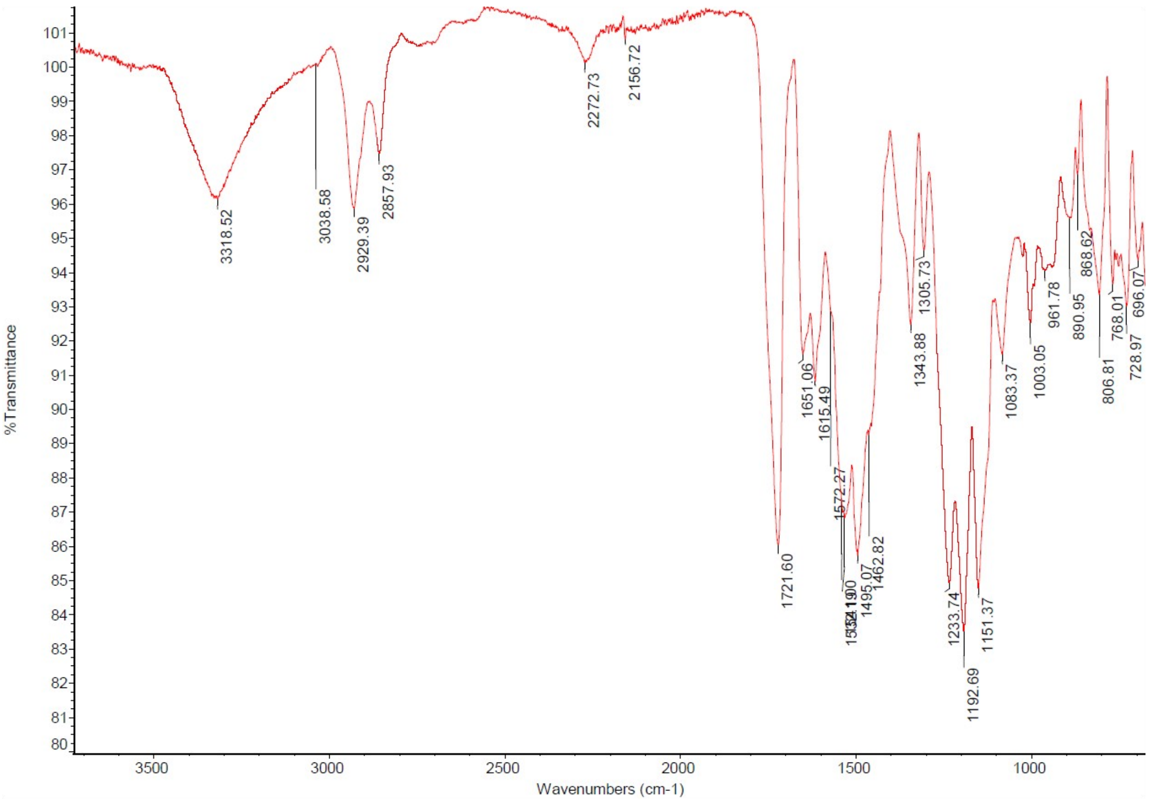

*Figure S4 IR spectrum of cross-linked **4**.*

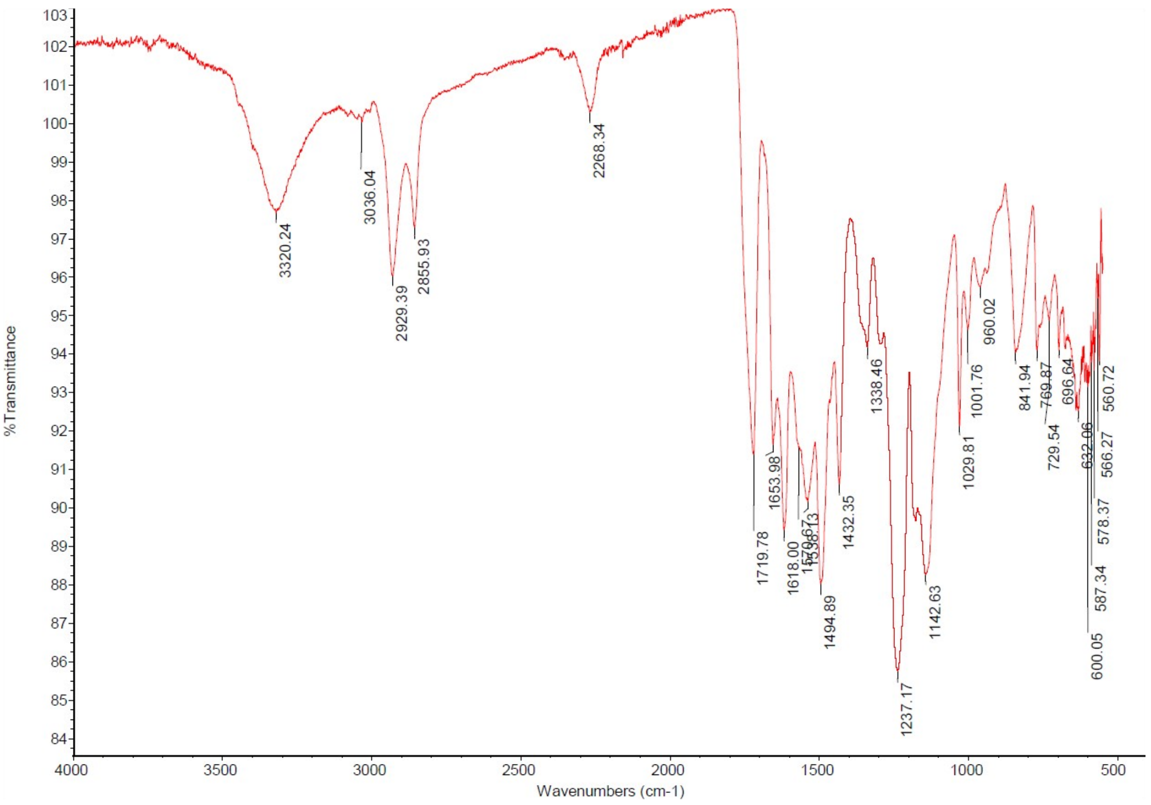

*Figure S5 IR spectrum of cross-linked **5**.*

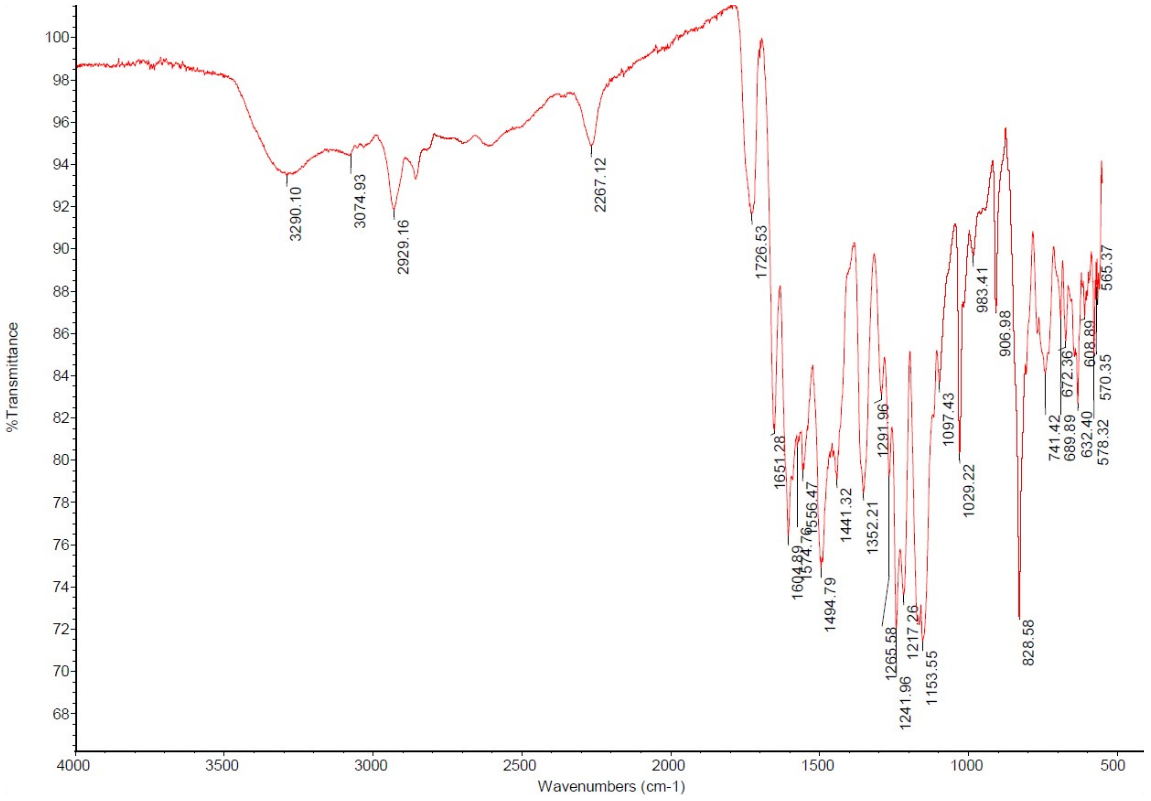

*Figure S6 IR spectrum of cross-linked **6**.*

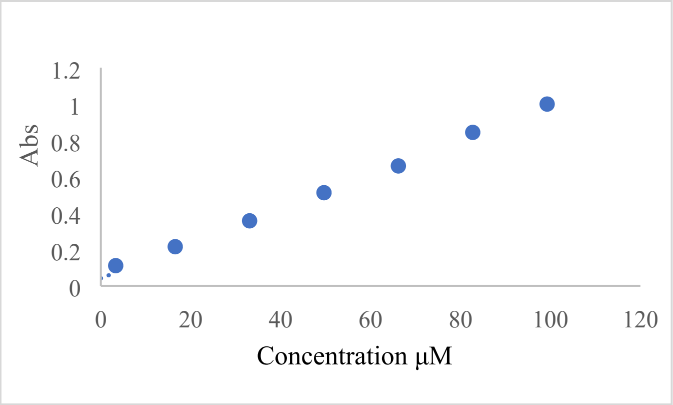

Figure S7 Quercetin standard concentration curve for release studies.

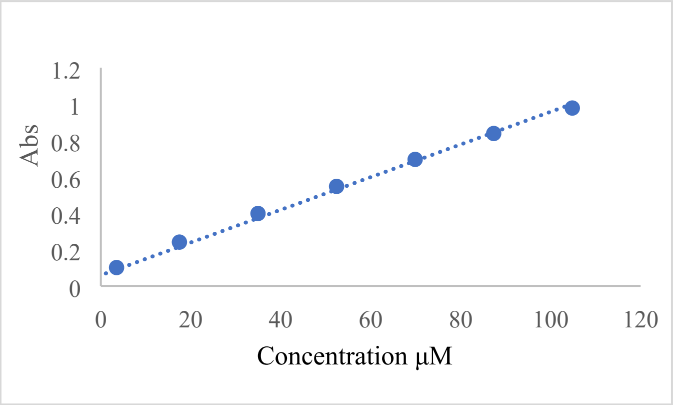

Figure S8 Luteolin standard concentration curve for release studies.

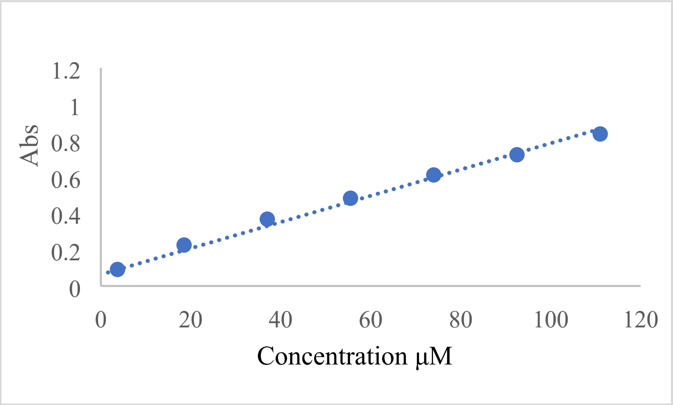

Figure S9 Apigenin standard concentration curve for release studies.

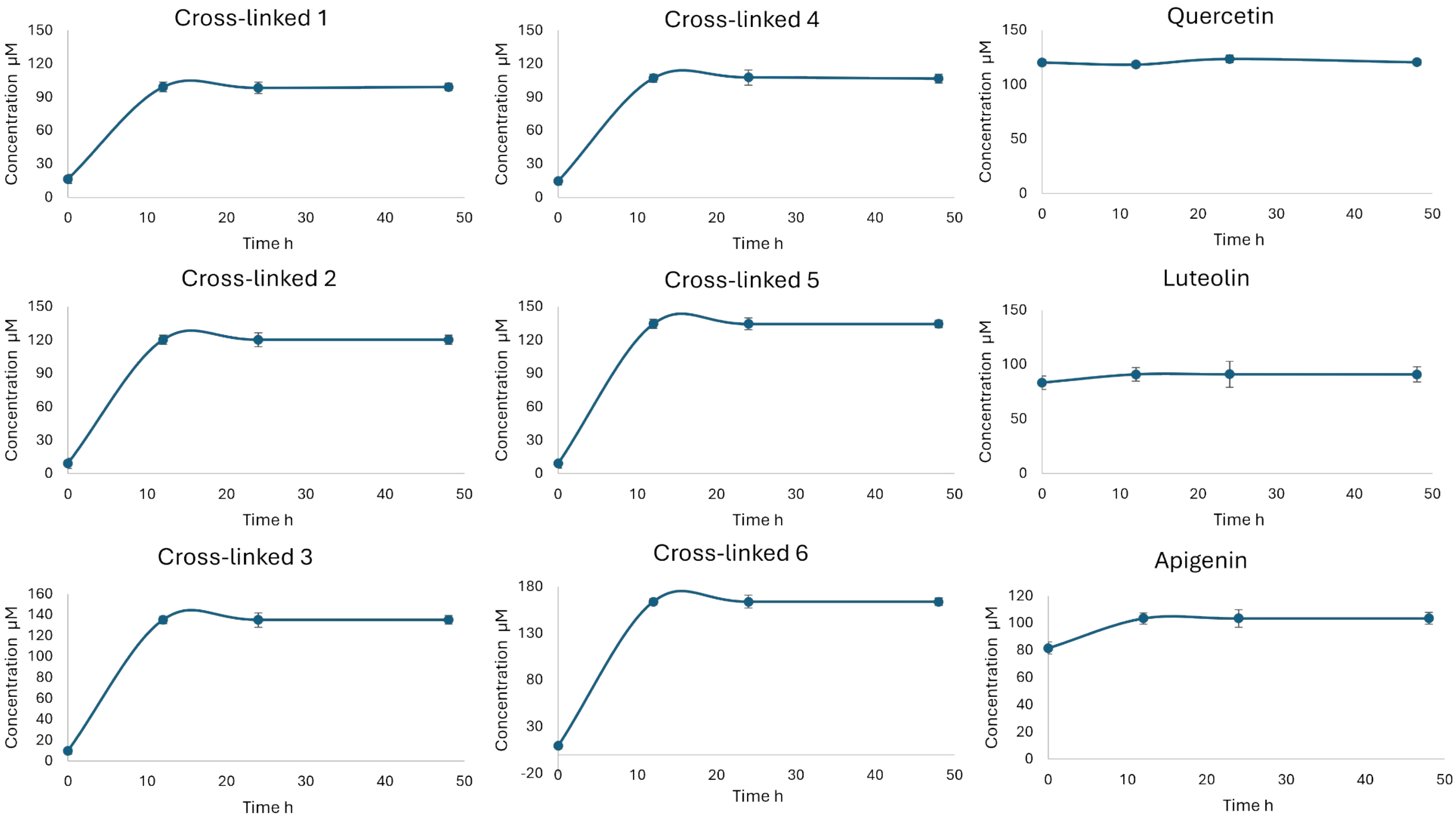

Figure S10 Determination of flavonoid content (wt/wt%) by exhaustive degradation and stability of the parent flavonoids under these conditions

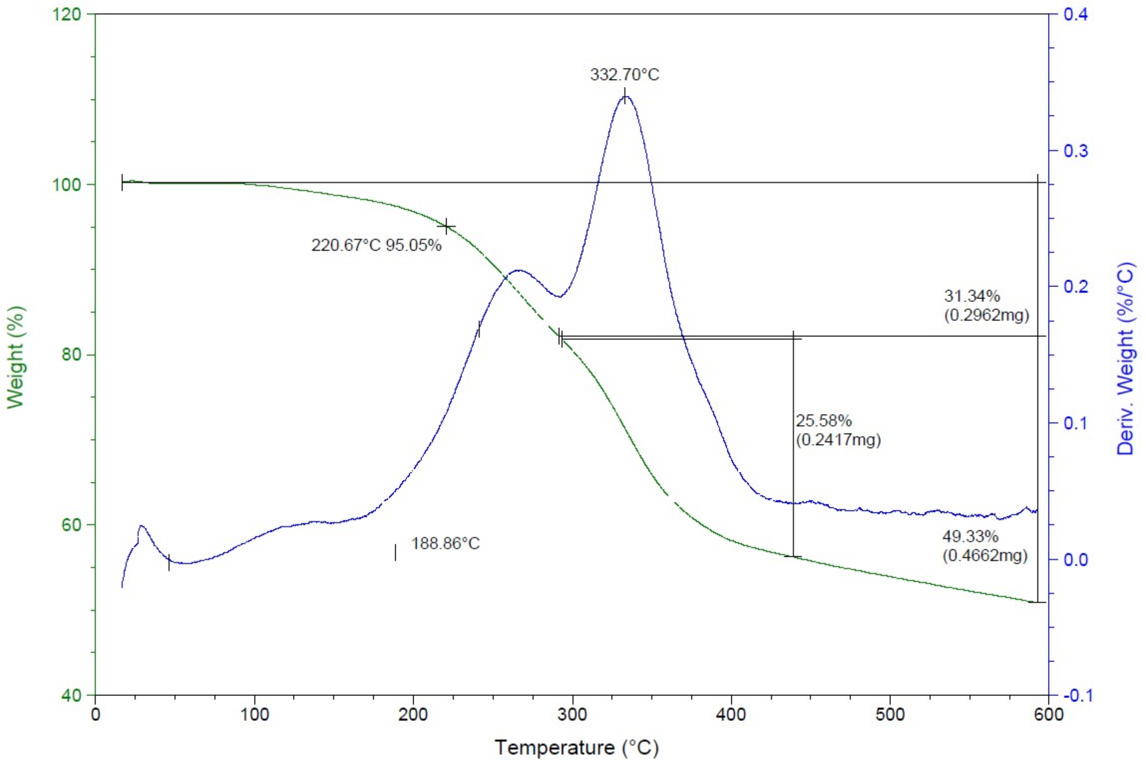

*Figure S11 TGA thermogram of cross-linked **1**.*

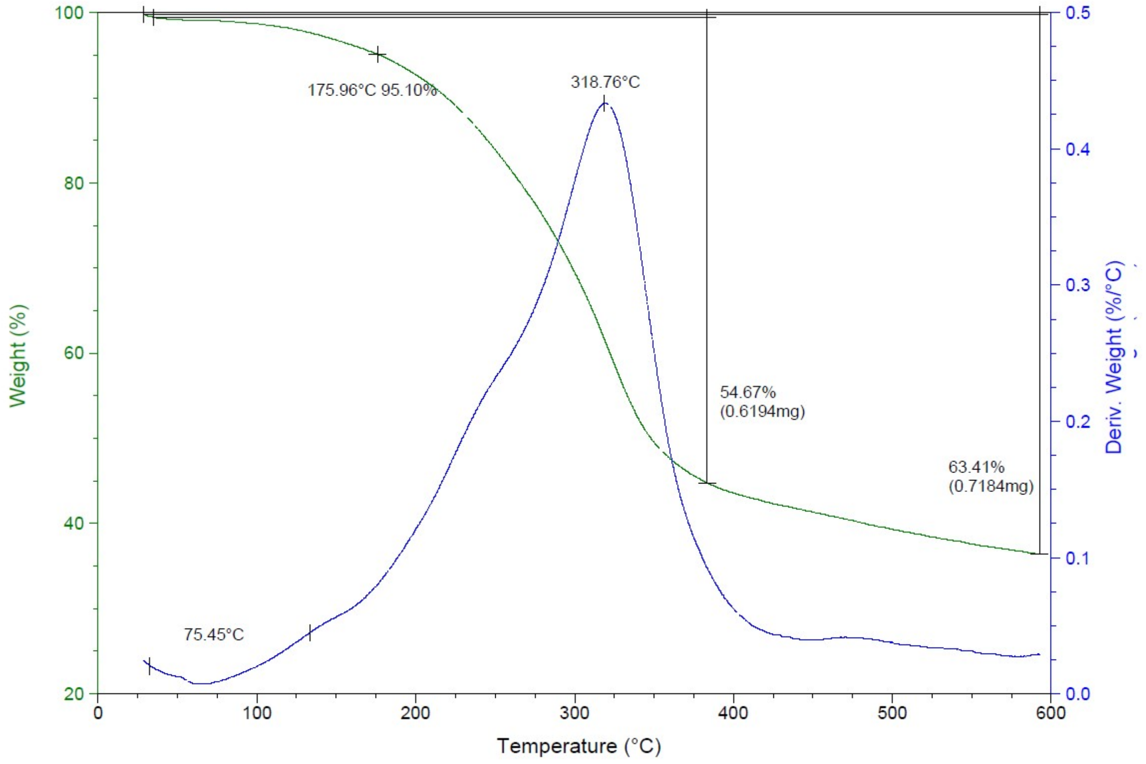

*Figure S12 TGA thermogram of cross-linked **2**.*

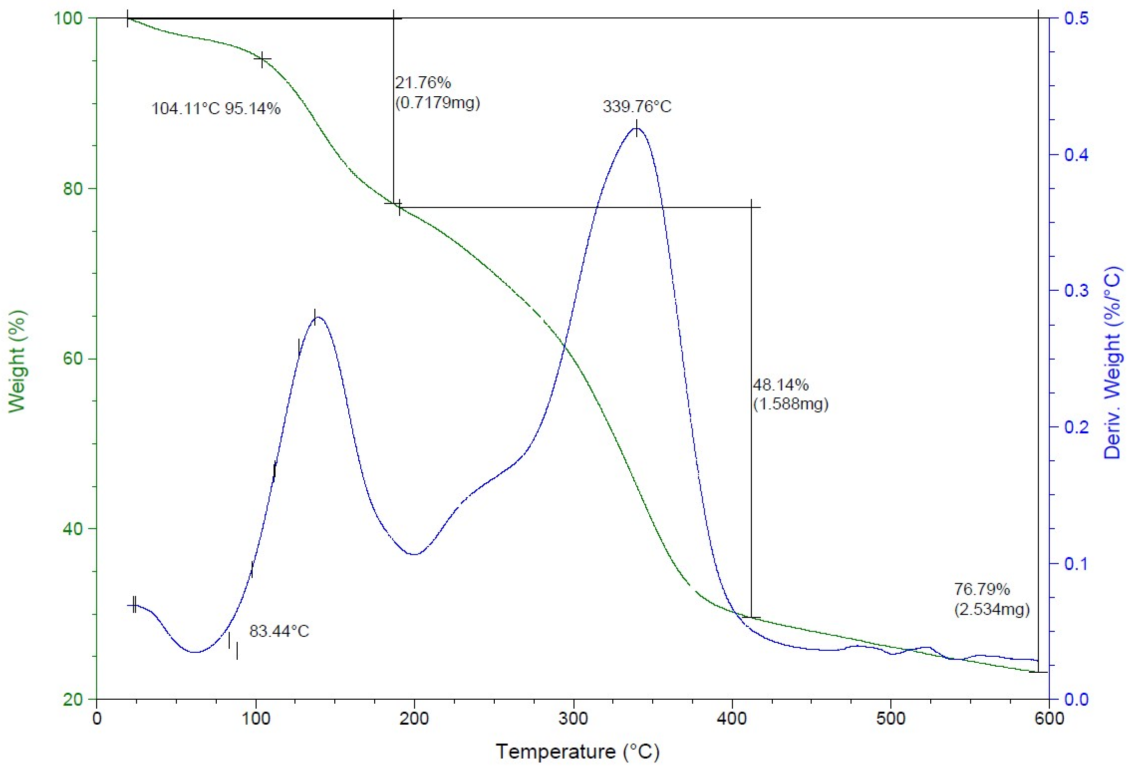

*Figure S13 TGA thermogram of cross-linked **3**.*

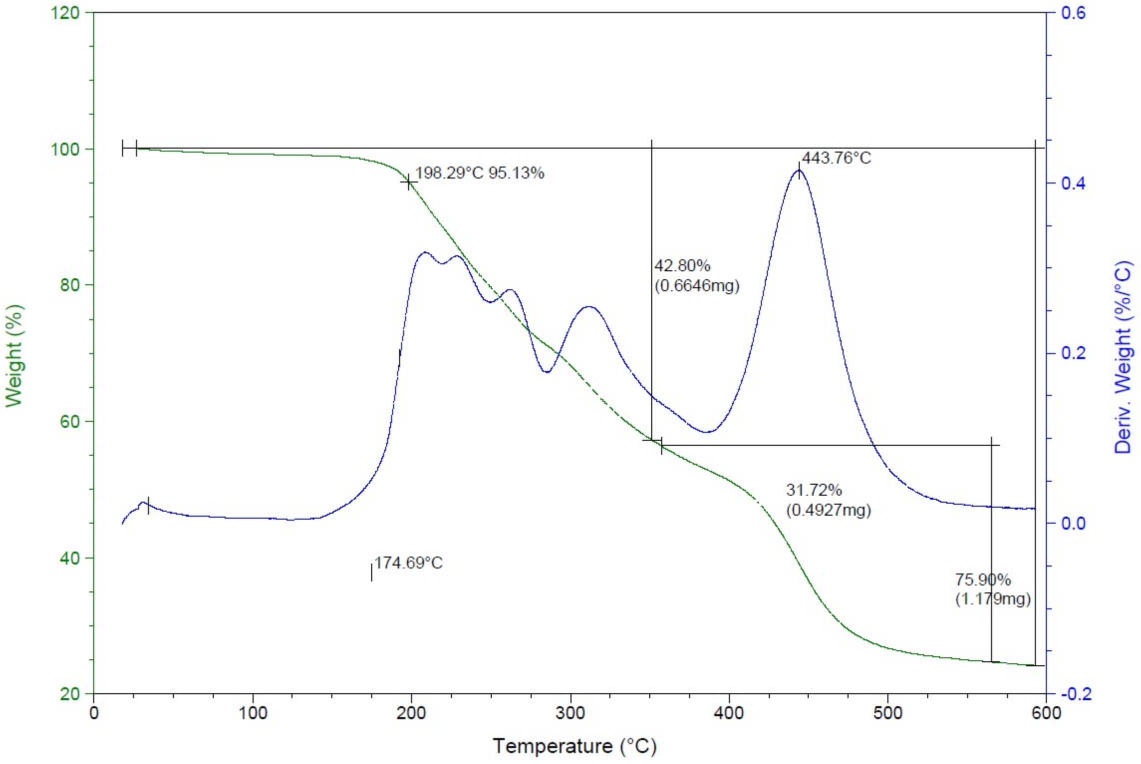

*Figure S14 TGA thermogram of cross-linked **4**.*

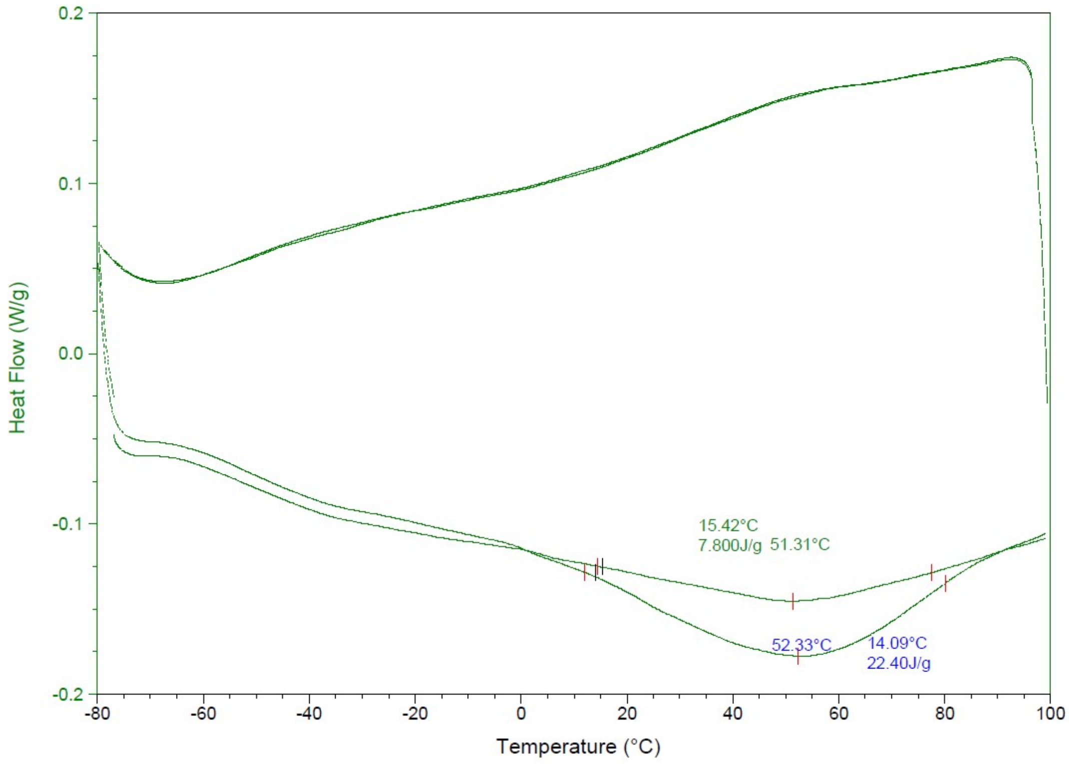

*Figure S15 DSC thermogram of cross-linked **1**.*

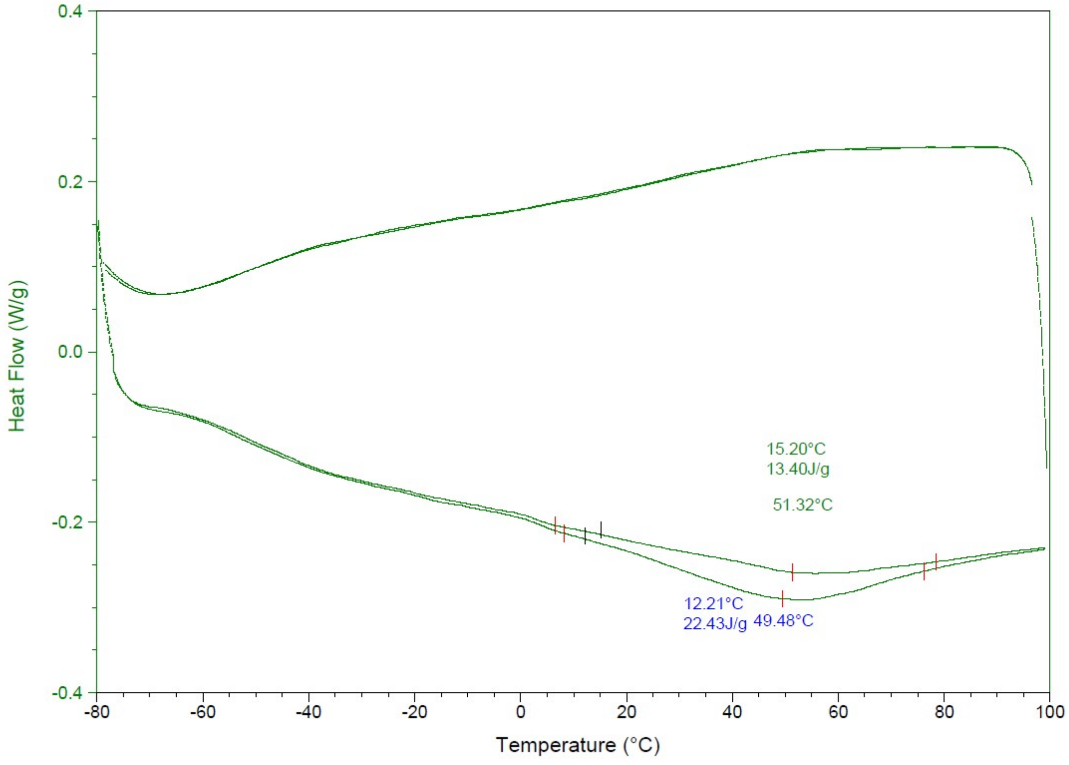

*Figure S16 DSC thermogram of cross-linked **2**.*

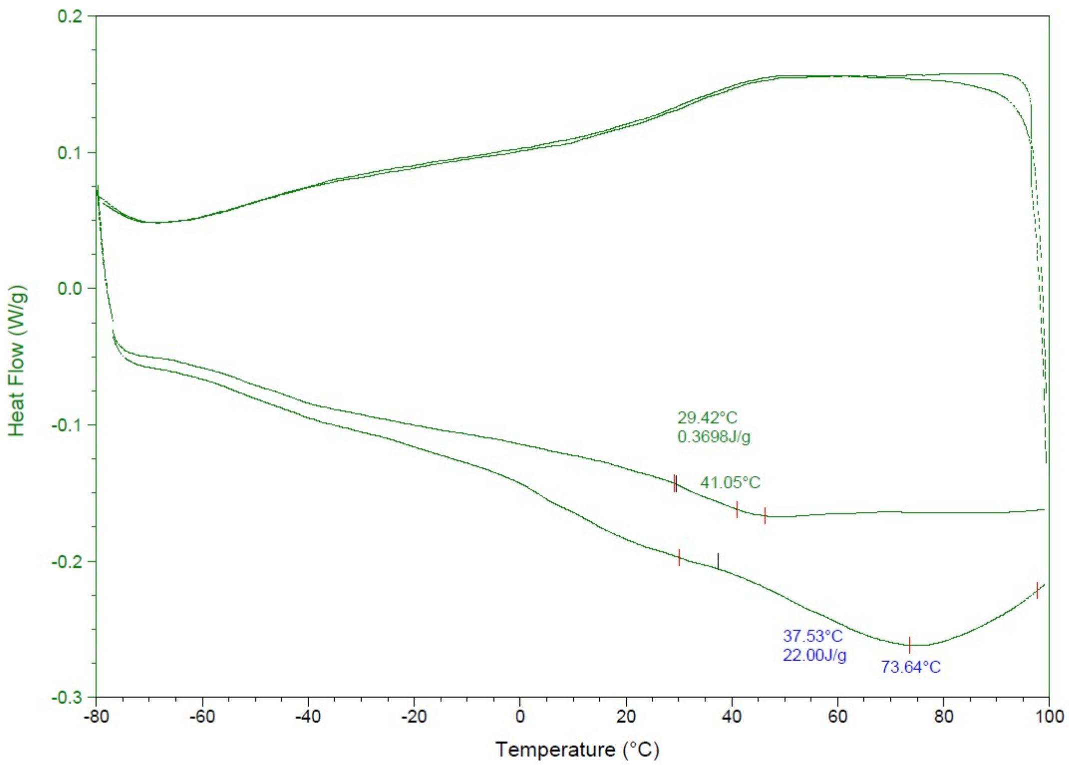

*Figure S017 DSC thermogram of cross-linked **3**.*

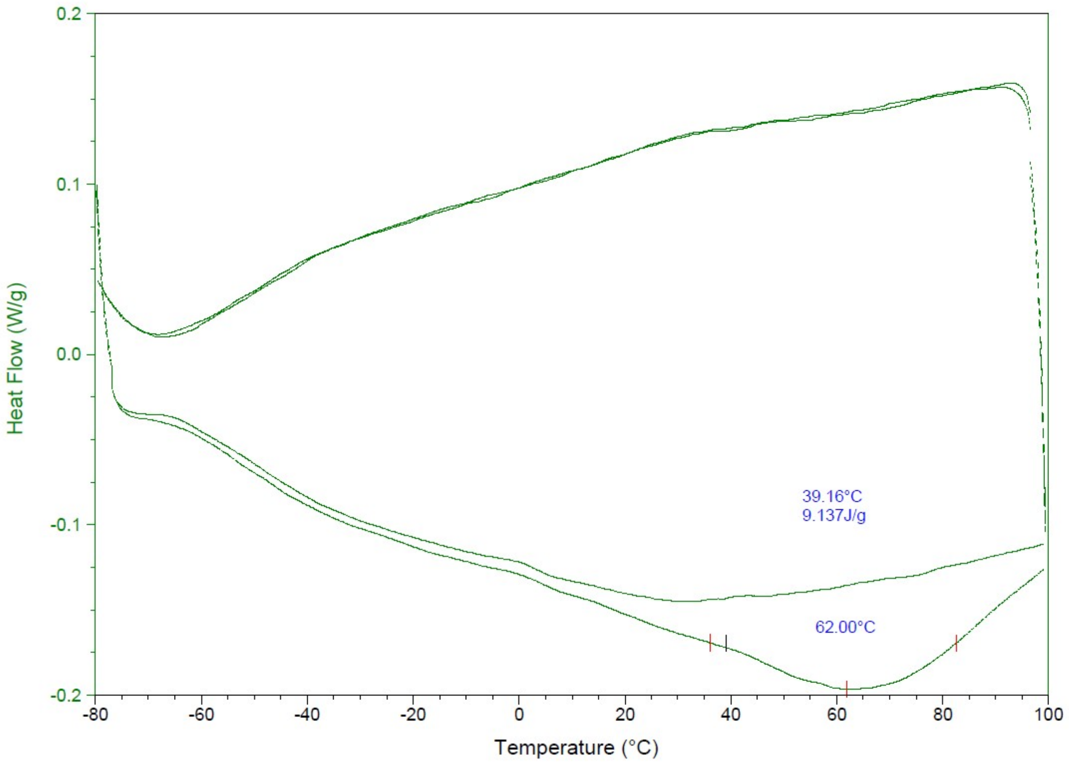

*Figure S18 DSC thermogram of cross-linked **4***

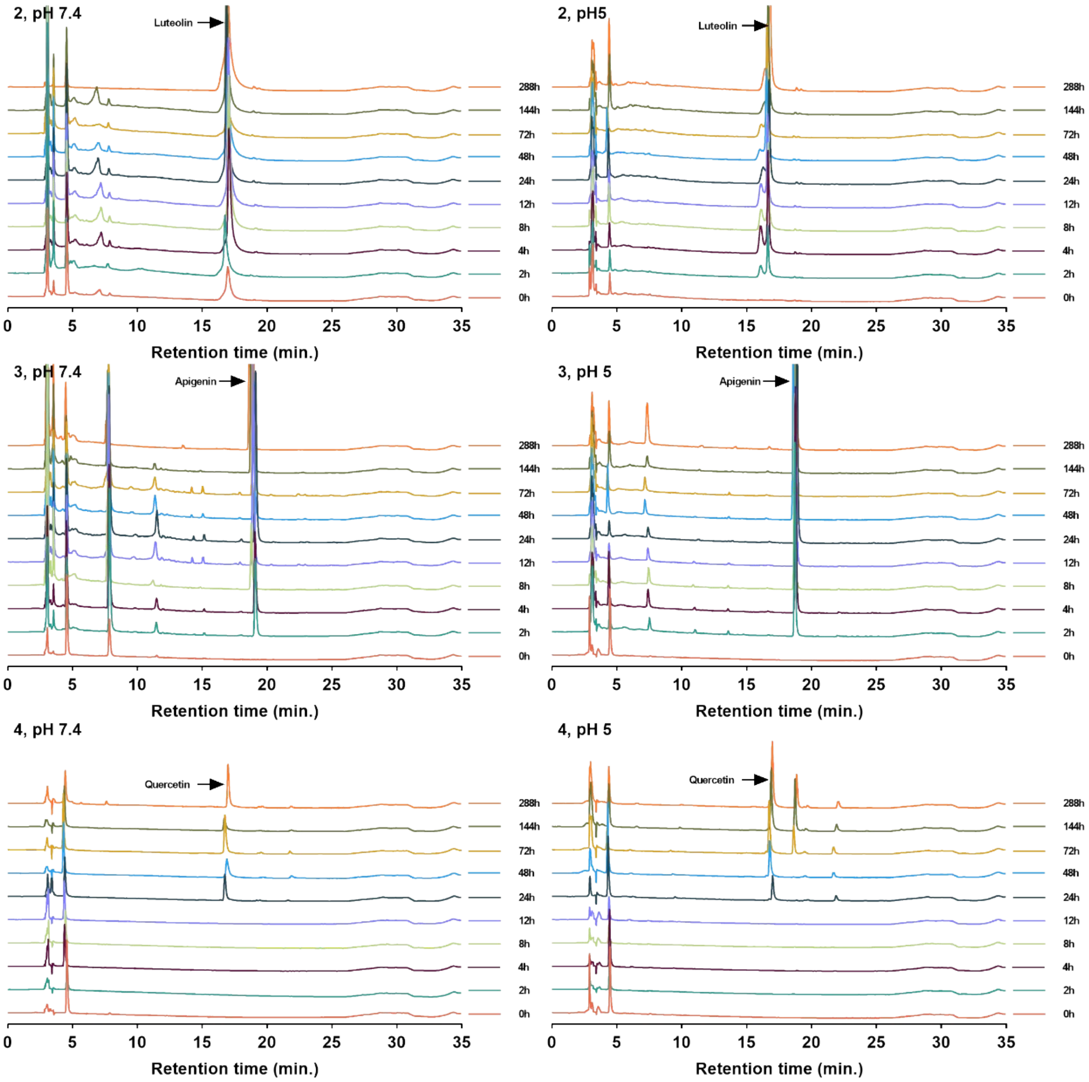
2, pH 7.4
Luteolin
2, pH5
Luteolin
288h
144h
72h
48h
24h
12h
8h
4h
2h
0h
0
5
10
15
20
25
30
35
Retention time (min.)
3, pH 7.4
Apigenin
3, pH 5
Apigenin
4, pH 7.4
Quercetin
4, pH 5
Quercetin

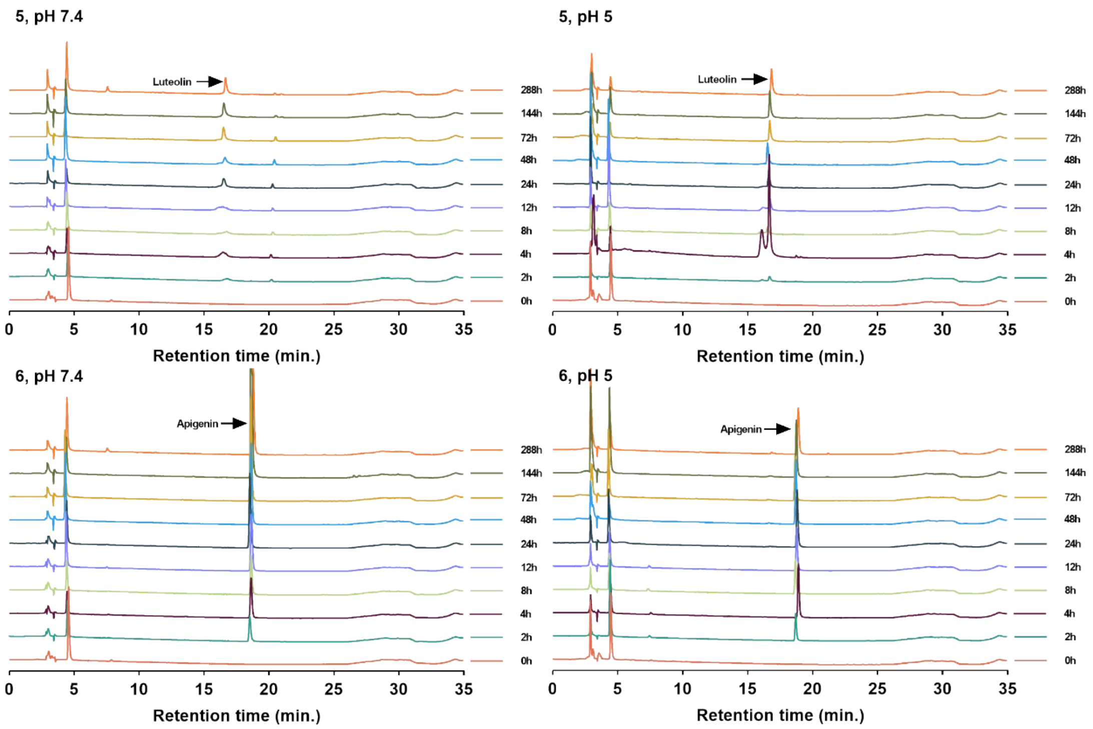

*Figure S19 HPLC chromatograms of quercetin, luteolin and apigenin release (in vitro) over 288 hours from cross-linked products **2,3,4,5,** and **6** incubated in PBS 7.4 or 5.*

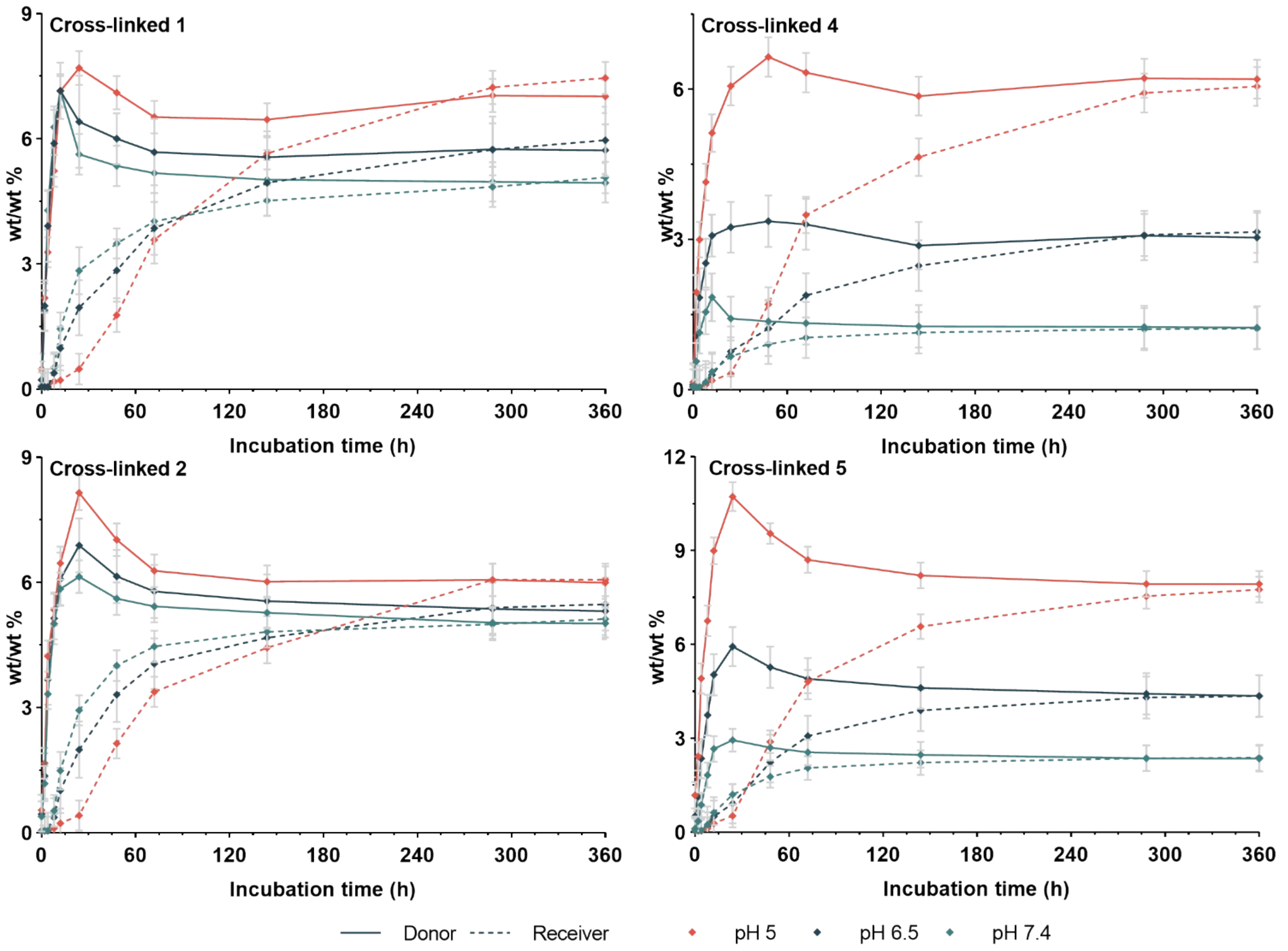

*Figure S20 Diffusion profiles of quercetin and luteolin release (in vitro) over 360 hours from the donor to the receiver chamber of the diffusion system from cross-linked products **1** and **4** respectively in pH 7.4 and pH 5. Data are expressed as mean ±SEM. (n=3)*

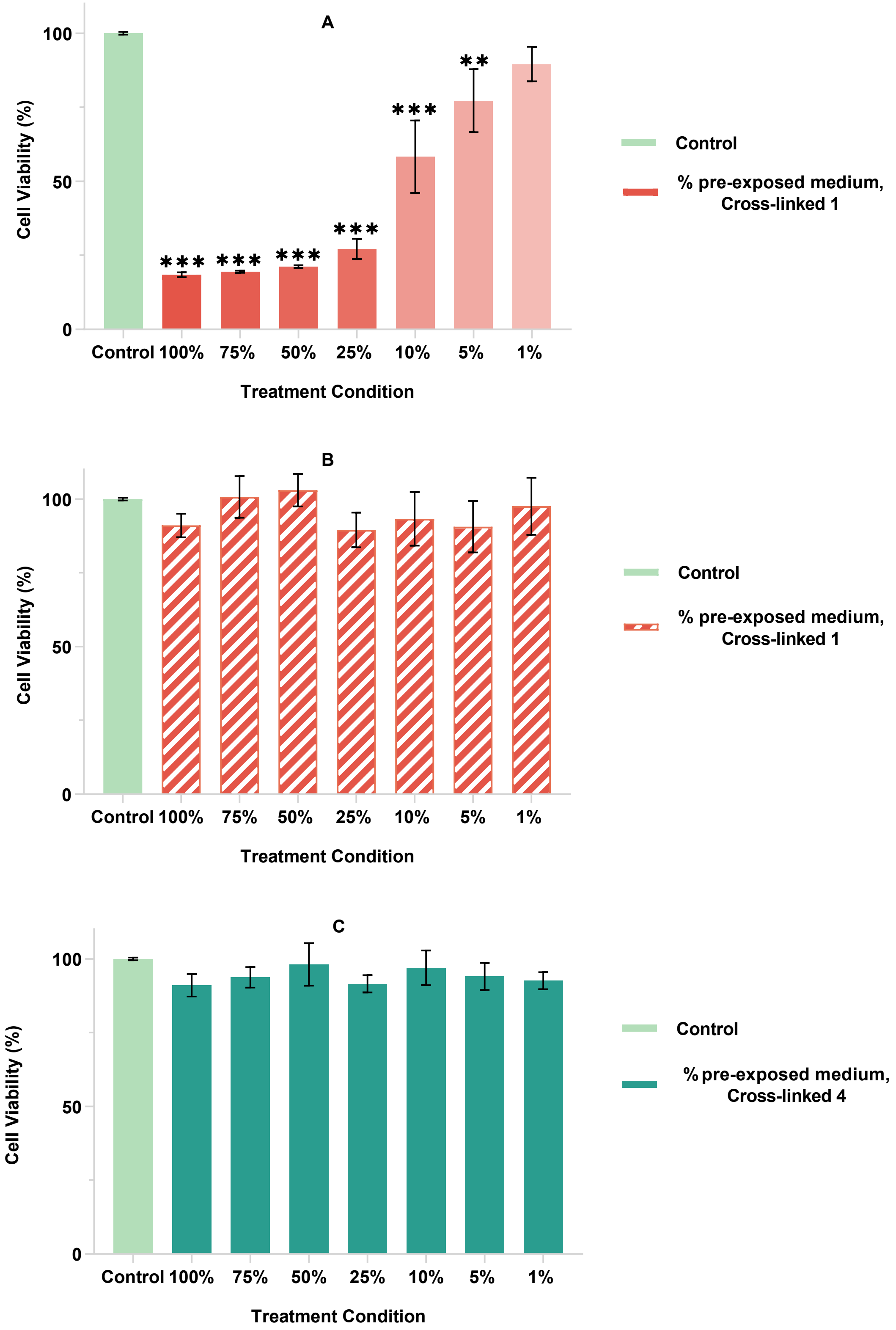
A
Cell Viability (%)
100
50
0
***
***
***
***
***
**
Control 100% 75% 50% 25% 10% 5% 1%
Treatment Condition
Control
% pre-exposed medium,
Cross-linked 1
B
Cell Viability (%)
100
50
0
Control 100% 75% 50% 25% 10% 5% 1%
Treatment Condition
Control
% pre-exposed medium,
Cross-linked 1
C
Cell Viability (%)
100
50
0
Control 100% 75% 50% 25% 10% 5% 1%
Treatment Condition
Control
% pre-exposed medium,
Cross-linked 4

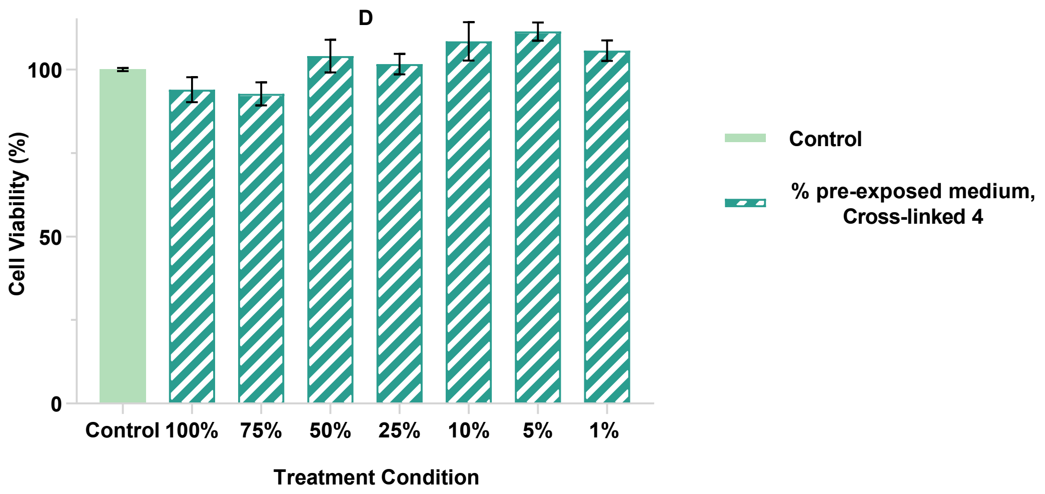

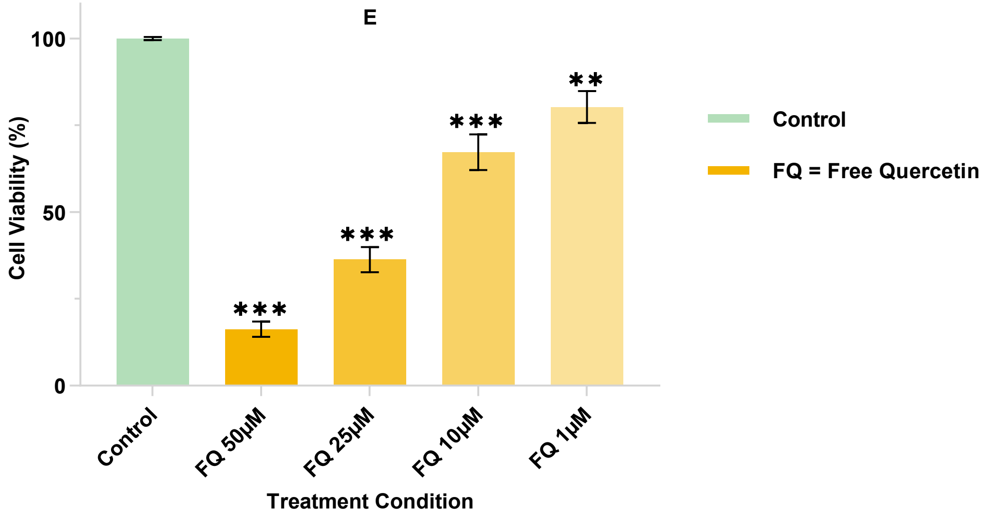

Figure S21 Anti-proliferative activities of a series dilutions releasing medium from cross-linked ***1*** *incubated for A) 24 hours and B) 5 minutes and cross-linked* ***4*** *incubated for C) 24 hours (solid bars) and D) 5 minutes (striped bars) compared to E) free quercetin standards, against MCF-7 cell line using an MTT assay. Data are expressed as mean ± SEM (n = 3).*

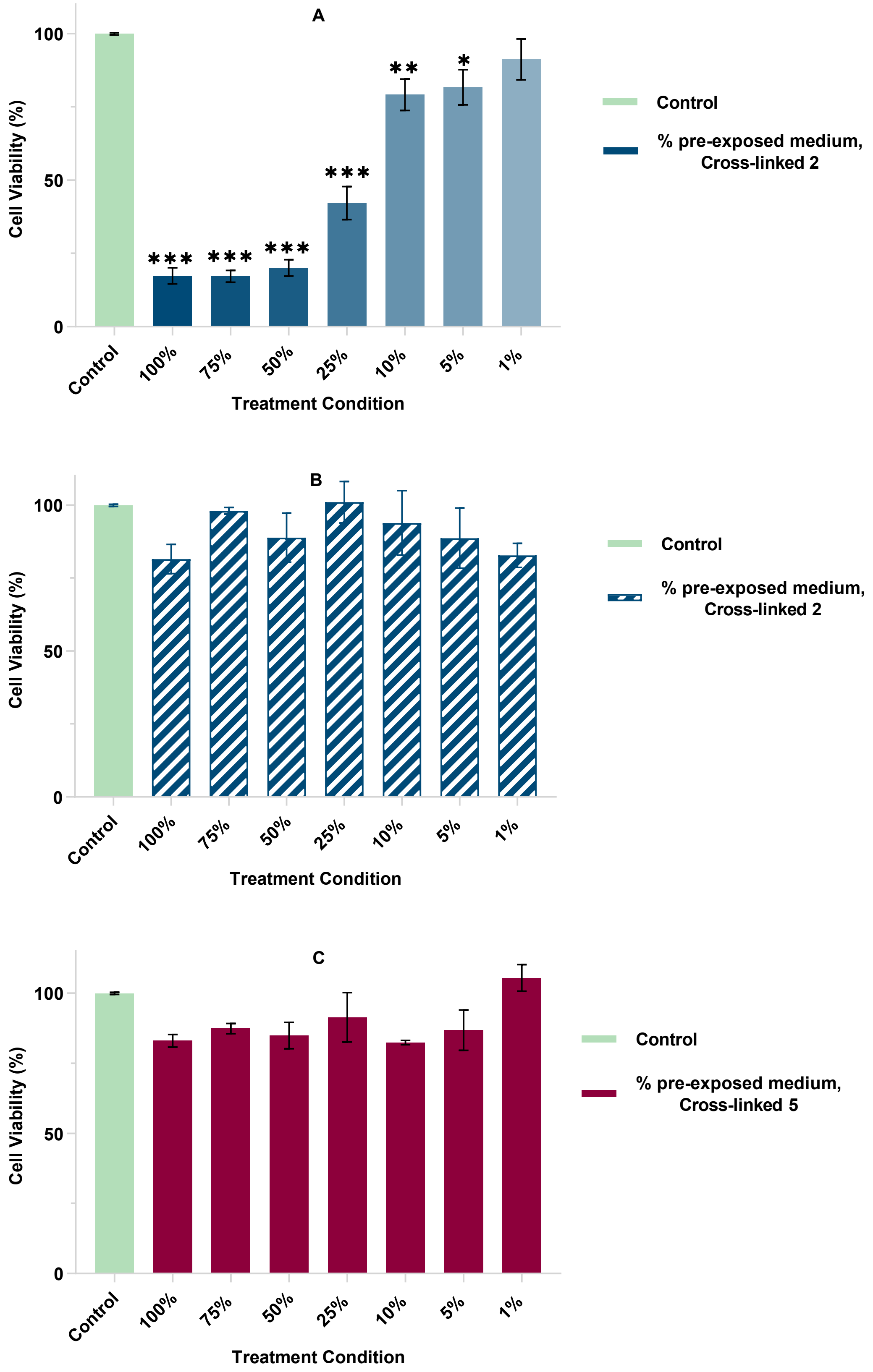
A
100
50
0
Cell Viability (%)
***
***
***
***
**
*
Control
100%
75%
50%
25%
10%
5%
1%
Treatment Condition
Control
% pre-exposed medium,
Cross-linked 2
B
100
50
0
Cell Viability (%)
Control
100%
75%
50%
25%
10%
5%
1%
Treatment Condition
Control
% pre-exposed medium,
Cross-linked 2
C
100
50
0
Cell Viability (%)
Control
100%
75%
50%
25%
10%
5%
1%
Treatment Condition
Control
% pre-exposed medium,
Cross-linked 5

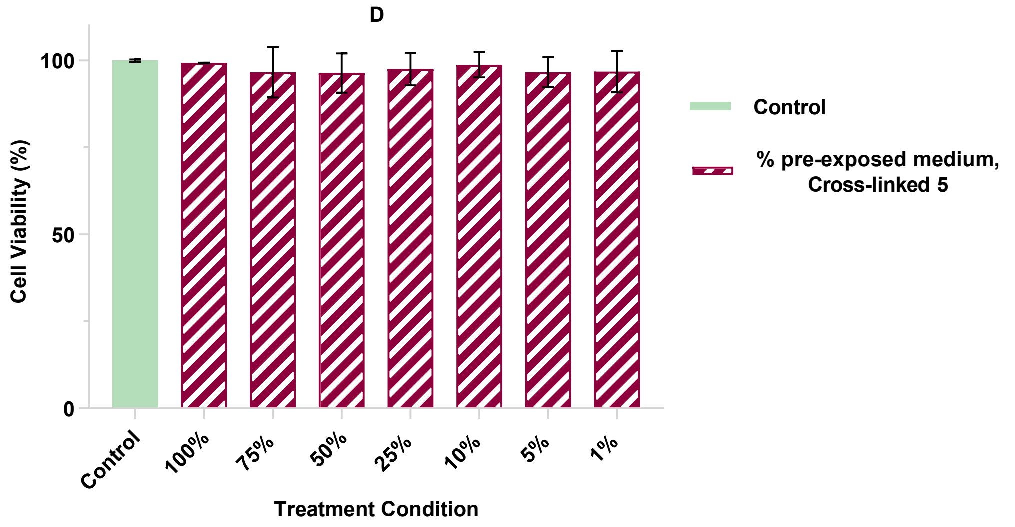

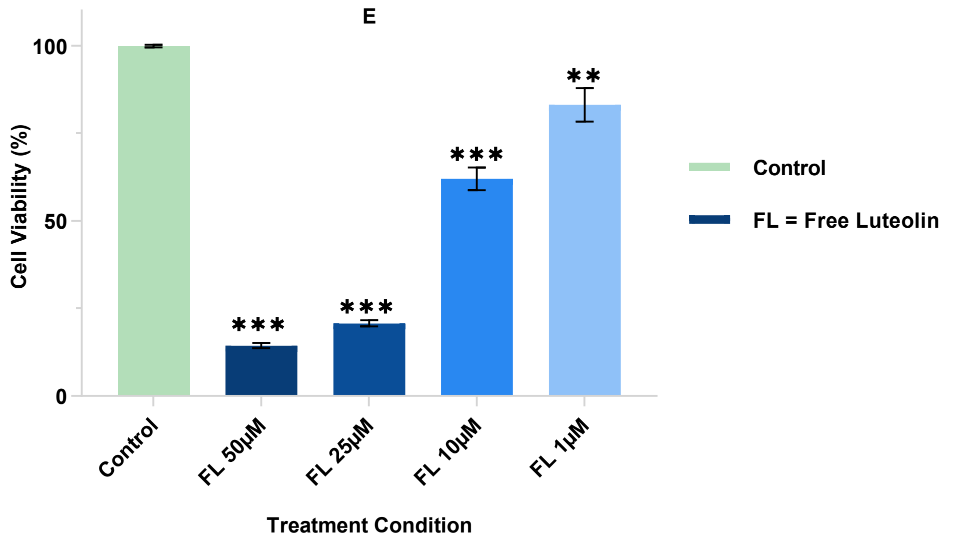

Figure S22 Anti-proliferative activities of a series dilutions releasing medium from cross-linked ***2*** *incubated for A) 24 hours and B) 5 minutes and cross-linked* ***5*** *incubated for C) 24 hours (solid bars) and D) 5 minutes (striped bars) compared to E) free luteolin standards, against MCF-7 cell line using an MTT assay. Data are expressed as mean ± SEM (n = 3).*

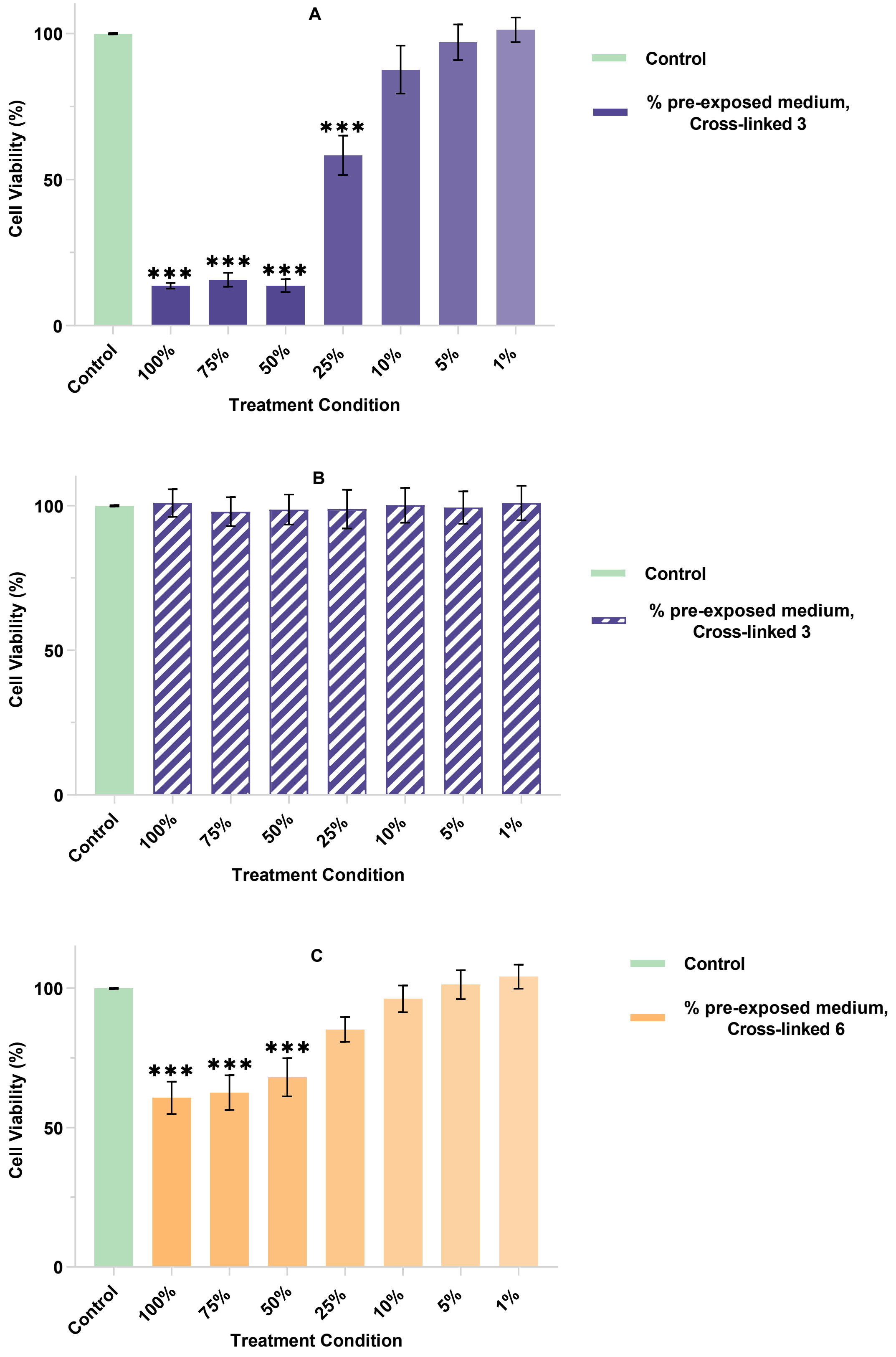
A
Cell Viability (%)
100
50
0
***
Control
100%
75%
50%
25%
10%
5%
1%
Treatment Condition
Control
% pre-exposed medium, Cross-linked 3
B
Cell Viability (%)
100
50
0
Control
100%
75%
50%
25%
10%
5%
1%
Treatment Condition
Control
% pre-exposed medium, Cross-linked 3
C
Cell Viability (%)
100
50
0
***
Control
100%
75%
50%
25%
10%
5%
1%
Treatment Condition
Control
% pre-exposed medium, Cross-linked 6

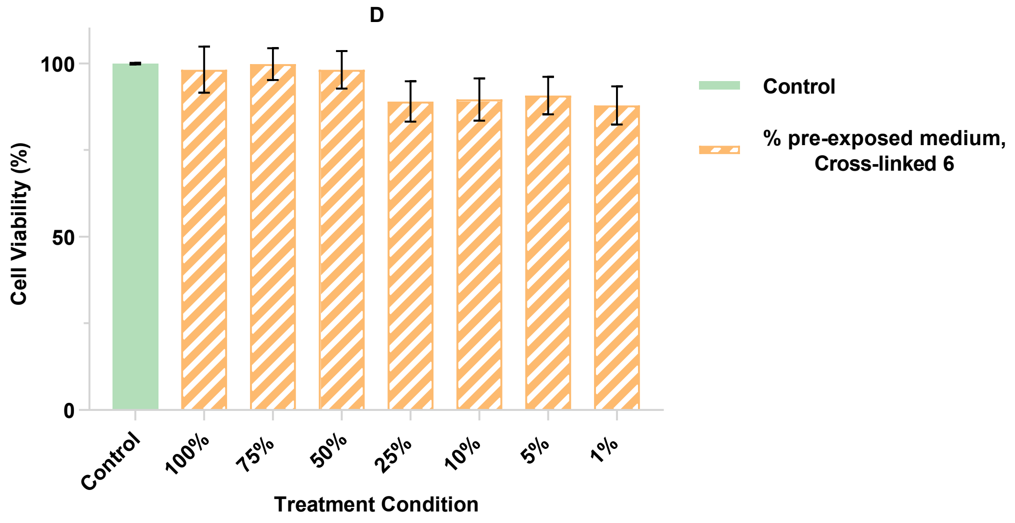

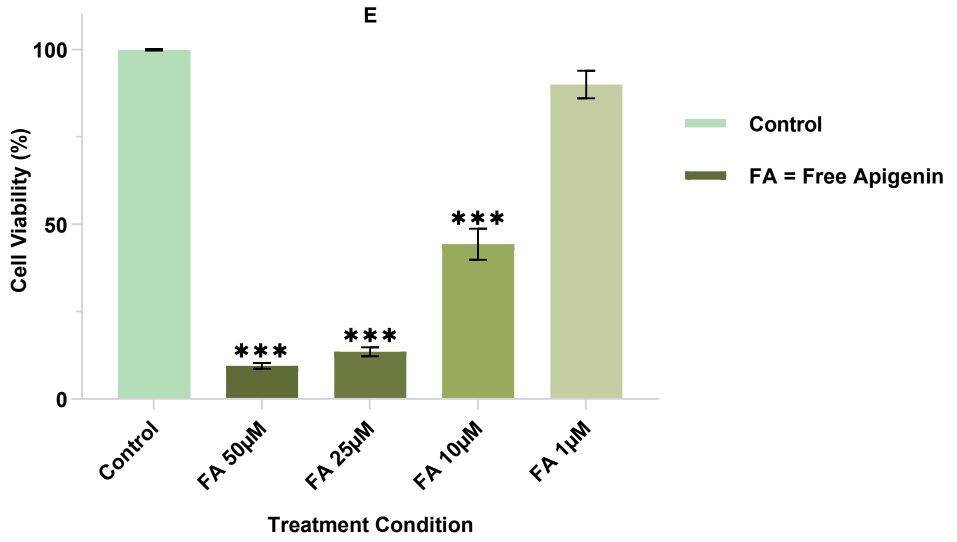

Figure S23 Anti-proliferative activities of a series dilutions releasing medium from cross-linked ***3*** *incubated for A) 24 hours and B) 5 minutes and cross-linked* ***6*** *incubated for C) 24 hours (solid bars) and D) 5 minutes (striped bars) compared to E) free apigenin standards, against MCF-7 cell line using an MTT assay. Data are expressed as mean ± SEM (n = 3).*

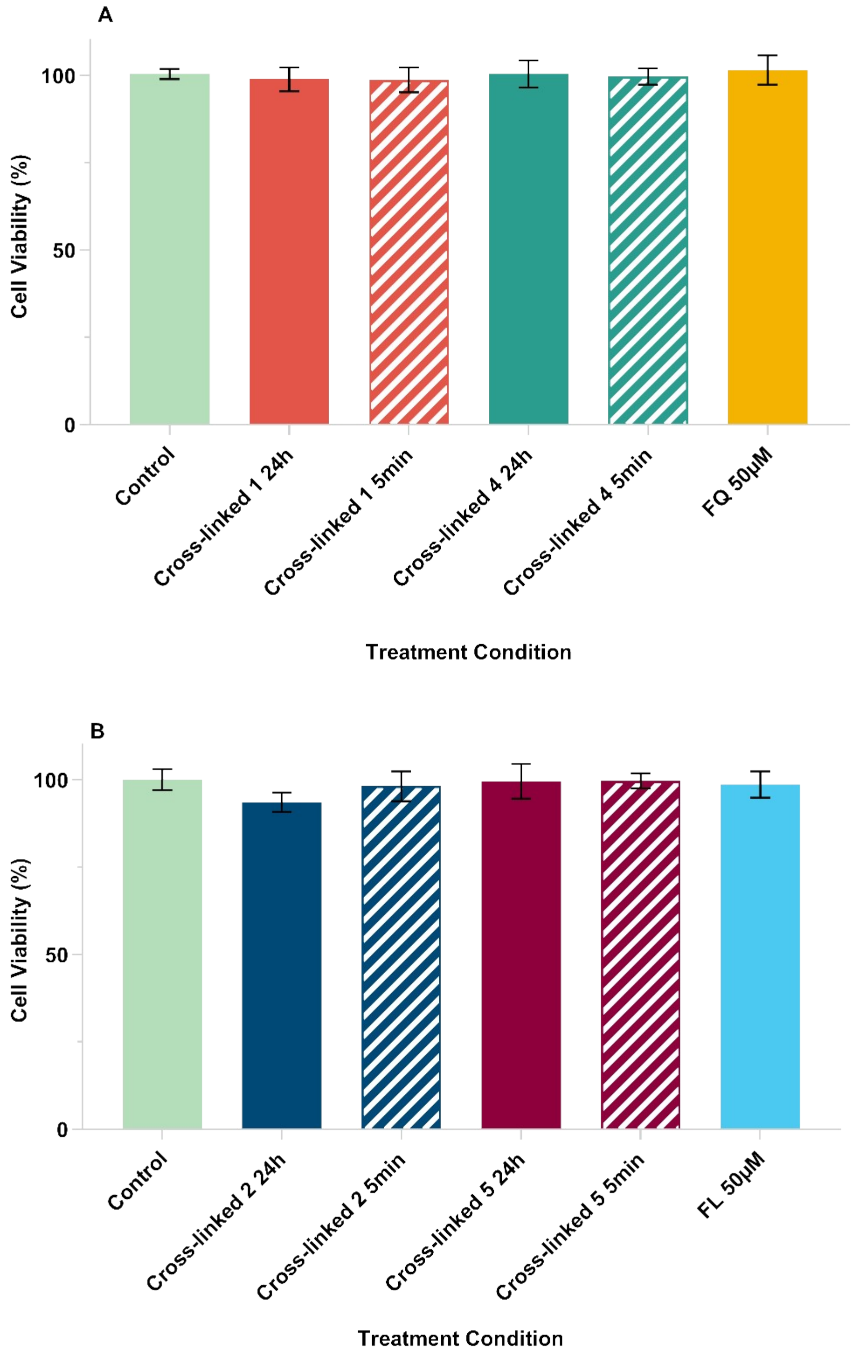
A
Cell Viability (%)
100
50
0
Control
Cross-linked 1 24h
Cross-linked 1 5min
Cross-linked 4 24h
Cross-linked 4 5min
FQ 50µM
Treatment Condition
B
Cell Viability (%)
100
50
0
Control
Cross-linked 2 24h
Cross-linked 2 5min
Cross-linked 5 24h
Cross-linked 5 5min
FL 50µM
Treatment Condition

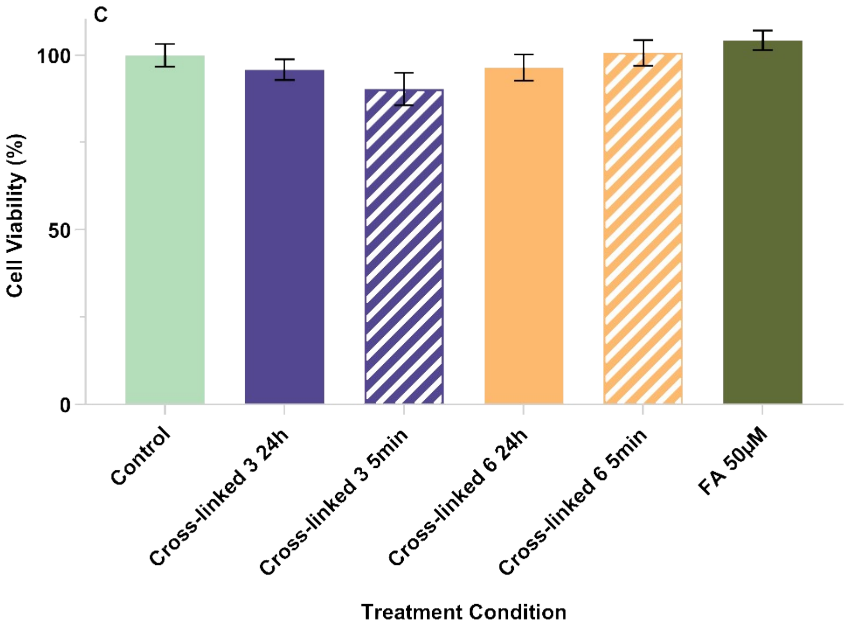

Figure S24 Anti-proliferative activities of undiluted (100%) liquid extracts from cross-linked networks, compared to 50 µM of free flavonoids, against 161BR cell line using an MTT assay. A) Cross-linked 1, 4 and free quercetin (FQ); B) Cross-linked 2, 5 and free luteolin (FL); C) Cross-linked 3, 6 and free apigenin (FA). Cell culture medium was pre-exposed to the cross-linked networks for 24 hours (solid bars) or 5 minutes (striped bars) prior to application. Data are expressed as mean ± SEM (n = 3).
